# Stallion Sperm Transcriptome Comprises Functionally Coherent Coding and Regulatory RNAs as Revealed by Microarray Analysis and RNA-seq

**DOI:** 10.1371/journal.pone.0056535

**Published:** 2013-02-11

**Authors:** Pranab J. Das, Fiona McCarthy, Monika Vishnoi, Nandina Paria, Cathy Gresham, Gang Li, Priyanka Kachroo, A. Kendrick Sudderth, Sheila Teague, Charles C. Love, Dickson D. Varner, Bhanu P. Chowdhary, Terje Raudsepp

**Affiliations:** 1 Department of Veterinary Integrative Biosciences, Texas A&M University, College Station, Texas, United States of America; 2 Department of Basic Sciences, Mississippi State University, Mississippi State, Mississippi, United States of America; 3 Department of Computer Sciences and Engineering, Mississippi State University, Mississippi State, Mississippi, United States of America; 4 Department of Large Animal Clinical Sciences, Texas A&M University, College Station, Texas, United States of America; University of Nevada School of Medicine, United States of America

## Abstract

Mature mammalian sperm contain a complex population of RNAs some of which might regulate spermatogenesis while others probably play a role in fertilization and early development. Due to this limited knowledge, the biological functions of sperm RNAs remain enigmatic. Here we report the first characterization of the global transcriptome of the sperm of fertile stallions. The findings improved understanding of the biological significance of sperm RNAs which in turn will allow the discovery of sperm-based biomarkers for stallion fertility. The stallion sperm transcriptome was interrogated by analyzing sperm and testes RNA on a 21,000-element equine whole-genome oligoarray and by RNA-seq. Microarray analysis revealed 6,761 transcripts in the sperm, of which 165 were sperm-enriched, and 155 were differentially expressed between the sperm and testes. Next, 70 million raw reads were generated by RNA-seq of which 50% could be aligned with the horse reference genome. A total of 19,257 sequence tags were mapped to all horse chromosomes and the mitochondrial genome. The highest density of mapped transcripts was in gene-rich ECA11, 12 and 13, and the lowest in gene-poor ECA9 and X; 7 gene transcripts originated from ECAY. Structural annotation aligned sperm transcripts with 4,504 known horse and/or human genes, rRNAs and 82 miRNAs, whereas 13,354 sequence tags remained anonymous. The data were aligned with selected equine gene models to identify additional exons and splice variants. Gene Ontology annotations showed that sperm transcripts were associated with molecular processes (chemoattractant-activated signal transduction, ion transport) and cellular components (membranes and vesicles) related to known sperm functions at fertilization, while some messenger and micro RNAs might be critical for early development. The findings suggest that the rich repertoire of coding and non-coding RNAs in stallion sperm is not a random remnant from spermatogenesis in testes but a selectively retained and functionally coherent collection of RNAs.

## Introduction

Mammalian sperm are considered terminally differentiated and functionally dormant cells with the sole purpose of delivering the paternal genome into the zygote [1], [2]. Therefore, early claims about the presence of RNA in mouse [3], bull [4], rat and human sperm [5] were met with skepticism. However, research over the past decade has provided compelling evidence that mature mammalian sperm contain complex populations of RNAs [1], [2], [6], [7], [8], [9]. These include over 3,000 mRNAs [6], [8], and a heterogeneous population of small and long non-coding RNAs [8], [10], [11], [12], [13], [14], though typically sperm are depleted of intact ribosomal RNAs [6].

The functions of sperm RNAs remain a subject of debate. The initial opinion was that sperm RNAs have no functions of their own and are simply residues of spermatogenesis, reflecting the events that occurred during their formation in the testes [1]. This may be partially valid, although recent discoveries have essentially expanded these views showing that sperm mRNAs constitute a population of stable full-length transcripts, many of which are selectively retained during spermatogenesis [6], [11]. Some mRNAs are thought to have a role in sperm chromatin reorganization by setting up boundaries between protamine- and histone-packaged DNA [11], [15]. Some mRNAs/cDNAs can be sperm-borne via transcription and reverse-transcription [10], [16]. It has been reported that sperm mRNAs can be *de novo* translated using mitochondrial-type ribosomes during capacitation [17], [18], [19]. Both sperm mRNAs and micro RNAs (miRNAs) are involved in non-Mendelian inheritance, serving as transgenerational epigenetic signals for zygotic gene regulation [20], [21], [22]. Furthermore, a few RNAs have been found only in the sperm and the zygote, but not in the oocyte, providing evidence for a unique paternal contribution [23], [24].

Even though the functions of the majority of the sperm RNAs remain enigmatic, it has been proposed that sperm transcriptional profiles might provide clinical markers for male fertility [1], [11]. Moreover, the non-invasive sample procurement through semen collection makes the approach particularly attractive. Indeed, an increasing number of studies in humans demonstrate that sperm mRNA profile can serve as a molecular diagnostic platform for evaluating male fertility [1], [9], [25], [26]. Consistent and biologically relevant qualitative and quantitative differences are present between the sperm RNAs of fertile men and men with abnormal reproductive phenotypes, such as skewed protamine ratios [27], teratozoospermia [26], cryptorchidism [28], reduced sperm motility [29], and idiopathic infertility [30], [31]. Similarly, sperm transcriptome studies have been initiated in bulls [29], [32], [33], [34] and boars [23], [35], [36] showing differences between the mRNA profiles of high- and low fertility bulls [34]. Analysis of porcine sperm, oocytes and two-cell embryos reveal that mRNAs of some genes, *viz*., *CLU*, *PRM1* and *PRM2* are delivered to the zygote exclusively by the sperm [23].

Despite the promising diagnostic potential of sperm RNAs for male fertility, the approach has found only limited attention in stallions [37], [38]. At the same time, poor fertility of breeding stallions is a recognized concern in the equine industry. While foal crop and stud fees form a principal component of the economy of the industry, stallions are typically selected on the basis of their ancestry and performance, and not for their reproductive potential [39]. As a result, about 36–43% of prospective breeding stallions do not pass the breeding soundness tests [40], [41].

The goal of this study was to obtain detailed information about the RNAs present in the sperm of normal fertile stallions to improve understanding of the biological significance of sperm RNAs and to establish a foundation for the discovery of sperm-based biomarkers for stallion fertility.

## Results

### Expression microarray analysis

Gene expression microarray analysis revealed 6,761 gene/EST transcripts in stallion sperm and 11,112 in the testes. The majority (97%) of the sperm transcripts were shared with the testes, while surprisingly, 165 transcripts were detected (at signal-to-noise ratio, SNR ≥2) only in the sperm and not in the testes, and are referred to as sperm-enriched transcripts.

Gene Ontology (GO) annotations were found for 3,319 (49%) sperm transcripts and grouped according to biological process (2,136; 78.9%), molecular function (1,503; 55.5%) and cellular component (2,270; 83.8%) (Table S1). The sperm transcripts were most significantly (p<0.001) involved in chemoattractant-activated signal transduction pathways, *viz.*, sensory perception and G-protein coupled signaling, and ion transport related biological processes. The most prevalent molecular functions were related to ion-, nucleotide-, and chromatin binding and the associated cellular components were membranes and vesicles (Table S2). These functional categories were also represented among the 165 sperm-enriched transcripts, though with lower significance values because of fewer genes analyzed (Table S3). In contrast, testes transcripts were localized in all cellular compartments and involved in diverse molecular functions and biological processes (data not shown).

**Figure 1:**
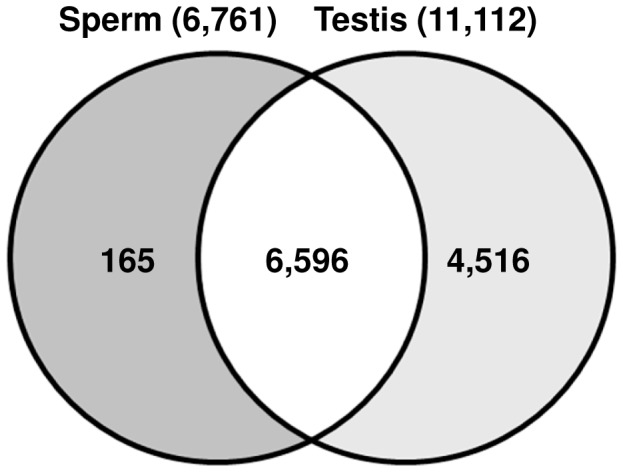
Venn diagram of transcripts detected in stallion sperm and testes by microarray analysis (SNR ≥2).

**Figure 2:**
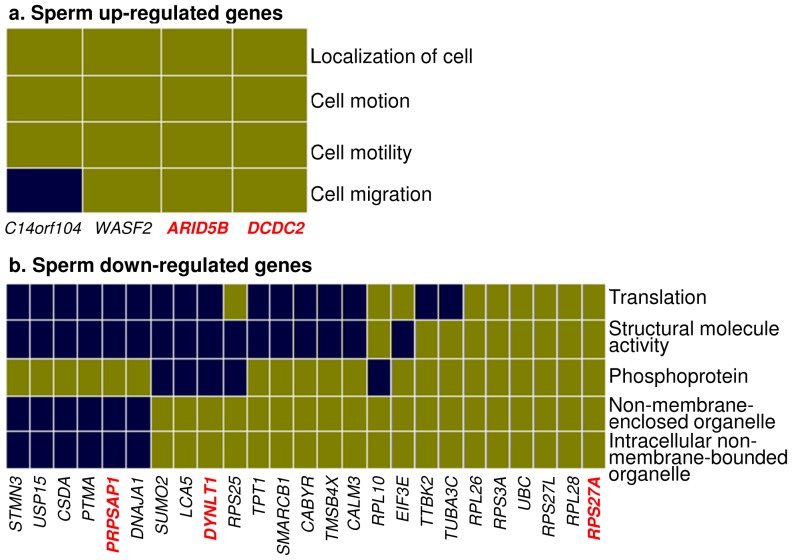
Heat maps of GO functional groups for sperm up-regulated (a) and sperm down-regulated (b) transcripts. Blue boxes denote that the gene has not been associated with the corresponding GO category. Genes with symbols in red font were validated by qRT-PCR.

**Figure 3:**
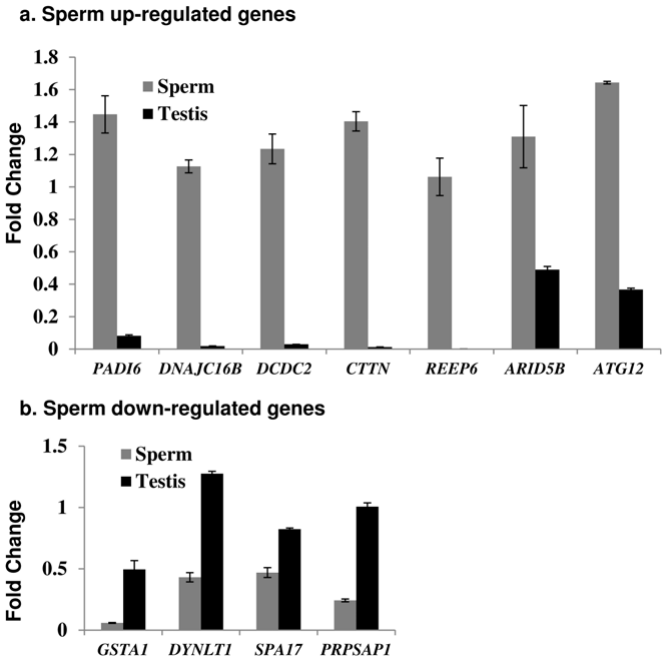
Validation of significantly (p<0.05) sperm up-regulated (a) and sperm down-regulated (b) genes by qRT-PCR (see also Table 1).

Comparison of the expression of the 6,596 transcripts common for the sperm and the testes (Fig. 1) identified 155 genes/transcripts that were differentially expressed (DE) between them. Of these, 60 were up-regulated (fold change; FC>2; p<0.05) and 95 were down-regulated (FC<−2; p<0.05) in the sperm (Table S4). Gene ontology terms could be determined for 37 up-regulated and 47 down-regulated transcripts showing that the former were involved in cell motility and cytoskeleton functions, while the latter were associated with functions in translation and non-membrane-enclosed organelles, e.g., ribosomes (Fig. 2). Microarray results for the most significant (p<0.005) DE genes were confirmed by quantitative RT-PCR (qRT-PCR) (Fig. 3; Fig. S1; Table 1).

**Table 1:** Selected most significantly (p<0.005) differentially expressed genes between stallion sperm and testes by microarray analysis and qRT-PCR (see also Fig. 3).

Gene symbol	DE	Primers for qRT-PCR 5′-3′	Function: GeneCards:http://www.genecards.org/
*PADI6*	up	F: GATTGTGATGGGCAAGAACC	Embryonic development
		R: AGCAGCTGGCAGATCTTTTC	
*DNAJC16B*	up	F: GAGAAGCAGGGCTACCAGAA	Unannotated
		R: TCTTTCCAAACAGGCTCGAT	
*DCDC2*	up	F: TGGCTTTTACCTGTGGGTCT	Sperm motility-sperm flagellum outer layer
		R: TACACCAGCACGCTCTTCAC	
*CTTN*	up	F: CTGGAACCCGTGTACAGCA	Sperm structure -organizing the cytoskeleton
		R: GCCTCCGTGCTTTCATAGAC	
*REEP6*	up	F: GGCTTCCTGTTGTTCTGCAT	Sperm structure-outer dense fiber of sperm tails
		R: CACTGCCTCGTGATGCTTTA	
*ARID5B*	up	F: GCTTGCACGGACCTTACATT	Regulation-regulator of smooth muscle cell differentiation and proliferation, defects in male reproductive organs; cryptorchid phenotype
		R: GGAGTGTCTTCTGGGAGGAA	
*ATG12*	up	F: TGGCAGTGATGGTAAACTGG	Regulation-bulk-protein degradation pathway, essential for autophagosomal formation
		R: CCACAAGTCTCTTGCCACAA	
*GSTA1*	down	F: GGAAGTTTGATGTTCCAGCA	Regulation-cytosolic and membrane bound- detoxification of electrophilic compounds
		R: GTATTTGGCGGCGATGTAGT	
*DYNTL1*	down	F: GCCTACCAGCACAGCAAAGT	Sperm structure -minus-end, microtubule-based motile processes
		R: TTAAACGGTTTTCCCAGCTT	
*SPA17*	down	F: TCCAAGGAGATGTCGATTCC	Surface protein -sperm surface zona pellucida binding protein
		R: GTTGCTCCCTCAGAATCTCG	
*PRPSAP1*	down	F: CTGATCATGGCTTACGCTCT	Regulation-negative regulation of 5-phosphoribose 1-diphosphate synthesis
		R: ATGGAACCCCTCTTCCTCAT	

### RNA sequence analysis

#### Mapping RNA sequence reads in the equine genome

**Figure 4:**
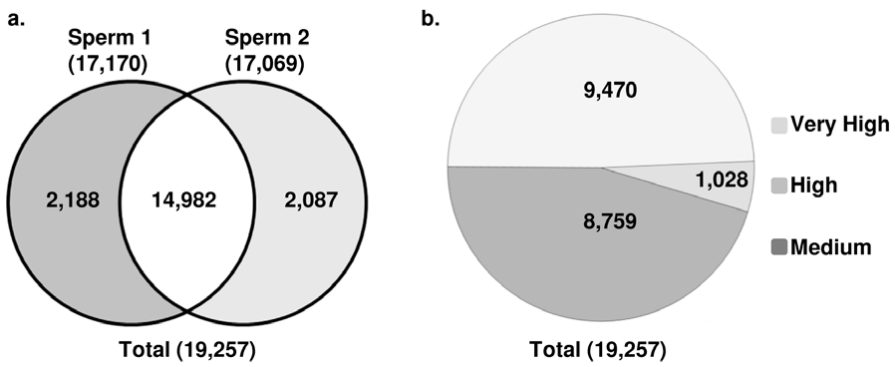
Summary statistics for mapped RNA sequence tags : (**a**) Comparison of mapped tags (AC≥1) between the two sperm samples; (**b**) Proportions of tags with very high (AC≥100), high (10<AC<100), and medium (1≤AC≤10) expression.

Next generation sequencing (NGS) of total RNA from the sperm of two reproductively normal stallions generated about 70 million raw reads and more than 3 Gb of sequence per sample; over half of these aligned with the EcuCab2 [42] reference genome (Table 2). Average coverage (AC; normalized number of transcripts) values could be calculated for over 30 million reads that mapped to all equine chromosomes, including ChrUn and the mitochondrial genome (Table 2, Table S9), whereas 19,257 sequence tags with AC ≥1 were uniquely mapped to specific locations in the horse genome (Table 2). Of these, 14,982 map locations were shared between the two samples, while 2,188 and 2,087 were unique to sample 1 and sample 2, respectively (Fig. 4a). These differences could be due to a combination of individual and technical variations, and justified the use of two biological replicates in this study. Genomic locations of all mapped tags together with their absolute and relative AC values are presented in Table S5. The data are deposited in NCBI Gene Expression Omnibus [43], [44] and are accessible through GEO series accession number *GSE38725* (http://www.ncbi.nlm.nih.gov/geo/query/acc.cgi?acc=GSE38725).

**Table 2:** Summary statistics for stallion sperm RNA-seq.

Number of:	Sperm 1	Sperm 2
Raw reads	64,488,380	76,386,416
Base-pairs	3,224,419,000	3,819,320,800
Reads aligned to EcuCab2	45,266,539	42,335,436
Alignment % to EcuCab2	70.2	55.4
Non-match reads	19,221841	34,050,980
Calculated total mapped reads*	38,087,876	30,261,556
Unique mapped reads**	17,170	17,069

* Aligned reads with calculated AC values.

** Reads with AC ≥1 mapped to unique locations (see Table S5 for details).

The 19,257 sequence tags mapped to all horse (*Equus caballus*, ECA) autosomes, the X chromosome, chromosome Un, and the mitochondrial (Mt) genome (Table 3). Because the horse Mt genome is only 16,660 bp [45], it showed the highest number of mapped tags per megabase (Mb) though only 3 tags mapped to this part of the genome. Among the autosomes, the number of tags in relation to chromosome size correlated well with the known gene densities and was the highest in ECA11, ECA12 and ECA13, and the lowest in ECA9 and ECAX (Table 3).

**Table 3:** Distribution and expression of mapped RNA sequence tags in the horse genome.

Sequence map data				RNA-seq data		
Horse chr.	Chr size, Mb	No. of genes*	Genes/Mb	No of tags(AC≥1)	Tags/Mb	ACmax
ECA1	185.8	2070	11.1	1,741	9	242,049
ECA2	120.8	1273	10.5	696	6	85,760
ECA3	119.4	1063	8.9	566	5	155,206
ECA4	108.5	980	9.0	390	4	30,327
ECA5	99.6	1221	12.3	440	4	15,063
ECA6	84.7	1107	13.1	346	4	31,927
ECA7	98.5	1455	14.8	412	4	49,977
ECA8	94	880	9.4	372	4	9,702
ECA9	94	610	6.5	276	3	7,597
ECA10	83.9	1204	14.4	1,451	17	18,607
ECA11	61.3	1245	20.3	1,544	25	49,866
ECA12	33	742	22.5	775	23	1,244
ECA13	42.5	745	17.5	1,023	24	4,197
ECA14	93.9	839	8.9	1,141	12	10,517
ECA15	91.5	815	8.9	1,159	13	42,457
ECA16	87.3	846	9.7	1,160	13	1,592
ECA17	80.7	497	6.2	754	9	4,436
ECA18	82.5	578	7.0	782	9	5,761
ECA19	59.9	541	9.0	574	10	2,189
ECA20	64.1	840	13.1	484	8	57,595
ECA21	57.7	491	8.5	290	5	1,717
ECA22	49.9	613	12.3	278	6	3,832
ECA23	55.7	410	7.4	281	5	14,218
ECA24	46.7	564	12.1	276	6	37,167
ECA25	39.5	605	15.3	284	7	12,795
ECA26	41.8	272	6.5	203	5	12,070
ECA27	39.9	277	6.9	217	5	5,922
ECA28	46.1	477	10.3	263	6	56,479
ECA29	33.6	246	7.3	141	4	55,336
ECA30	30	218	7.3	127	4	1,233
ECA31	24.9	176	7.1	116	5	19,300
ECAX	124.1	1239	10.0	390	3	133,493
ECAUn	n/a	n/a	n/a	302	n/a	272,950
Mt	0.016	37	2312.5	3	188	4,390
**TOTAL**				**19,257**		

**Mb**–megabase-pair; **AC**–average coverage; **Mt**–mitochondrial genome; map information for horse chromosomes was retrieved from Ensemb (http://www.ensembl.org/index. html); * includes known and novel protein coding, miRNA, rRNA, snRNA, snoRNA and Misc RNA genes.

According to the AC value which was used as the measure of expression level, the 19,257 tags fell into three categories: i) 1,028 (5%) tags with very high expression and AC≥100; ii) 8,759 (45%) tags with high expression and AC between 10 and 100, and iii) 9,470 (50%) tags with medium expression and AC between 1 and 10 (Fig. 4b). The distribution of very highly expressed tags in the horse genome was not uniform and tags with ACmax>100,000 were predominantly found in ECAUn, ECA1, ECA3, and ECAX, possibly indicating the locations of functionally important genes for the sperm. However, accumulation of very highly expressed tags to ECAUn is more likely because it contains multicopy sequences encoding for 18S and 28S ribosomal RNA which form about 80% of raw reads (see below). Compared to this, the ACmax in ECA12, ECA16 and ECA30 was less than 1,000 sequence reads per locus (Table 3).

Overall, there was a good agreement between the two sperm samples regarding the number and AC values of about 80% of mapped tags across the genome, including the tags with very high expression (Table S5). The most pronounced differences were cases where the same tag scored high or very high (AC>10) in one sample and low (AC<1) in another. Some differences in alignment of data in biological replicates were likely due to sequencing errors and chance alignments which is a significant problem for short reads and low alignment scores [46]. Among the 19,257 tags, 22% fell into this category and were uniquely mapped in sperm 1 or sperm 2 (Fig. 4a; Table S5).

#### Structural and functional annotation of RNA sequence data

**Figure 5:**
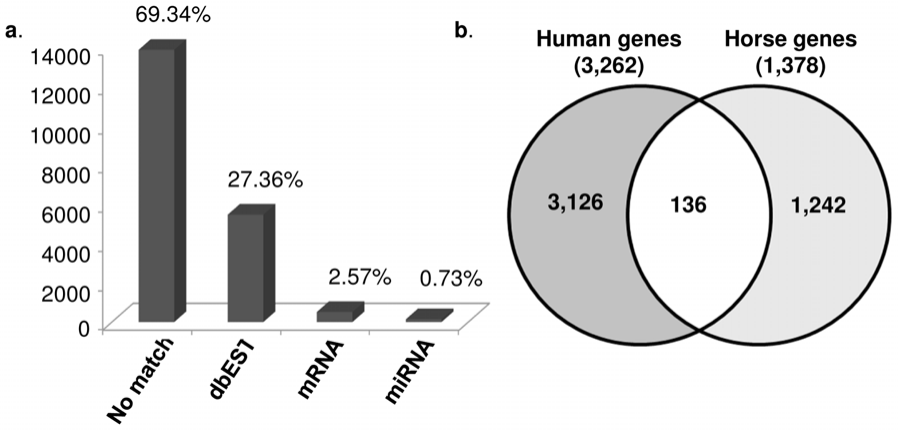
Structural annotation of 19, 257 mapped RNA sequence tags (AC≥1): (**a**) Distribution of the tags in structural annotation categories by ERANGE; (**b**) Comparison of annotated genes by GOanna (human genome) and ERANGE (horse genome).

Structural and GO annotations of the 19,257 mapped RNA-seq tags with AC≥1 were conducted by alignment to the equine reference sequence (EcuCab2; UCSC Genome Browser; http://genome.ucsc.edu/) using Enhanced Read Analysis of Gene Expression (ERANGE) software packages [47], as well as by homology-based approach against the human genome in GOanna (AgBase; http://www.agbase.msstate. edu/cgi-bin/tools/GOanna.cgi) pipeline. A total of 5,903 (∼30%) of all mapped tags, aligned with annotated genes in the horse genome and were classified by ERANGE as expressed sequence tags (5,268), mRNAs (495) and micro RNAs (140) (Fig. 5a). Since the structural annotation of the equine genome is as yet incomplete, we used a permissive ±20 kb parameter to identify additional untranscribed regions (UTRs), new external exons, and to discriminate best candidates for novel genes. Among the 5,903 annotated transcripts, ∼17% entirely fell within the boundaries of annotated genes, 83% partially aligned with known genes, and 0.03% localized within the extended gene boundaries (see Materials and Methods). Only 1,378 annotations uniquely corresponded to individual equine genes. Similarly, 34% (6,606) of all mapped RNA-seq tags aligned with annotated sequences in the human genome identifying 3,262 unique genes. Because the horse (ERANGE) and human (GOanna) annotations shared only 136 genes in common (Table S6), stallion sperm transcripts as observed by RNA-seq analysis corresponded to a total of 4,504 annotated genes (Fig. 5b).

The majority of mapped RNA sequence tags (13,354 tags, 70%) had no match in the current horse genome draft assembly by ERANGE. From the tags that could not be annotated, we selected 12 tags with extremely high average coverage values (AC>50,000) and showed by manual BLAST analysis that 67% of these tags were highly similar to the rRNA in the 60S (5S, 28S) and 40S (18S) subunits of the eukaryotic ribosome (Table 4). High AC values of these tags indicated abundant representation of rRNA in stallion sperm.

**Table 4:** NCBI BLAST alignments for 12 most abundant (AC>50,000) un-annotated mapped RNA sequence tags.

Chromosomal Location	AC	NCBI BLAST alignment	NCBI accession number	E-value	Identity %
chrUn:41671499-41671648	272,950	*Sus scrofa* 28S rRNA	AB117610	2E-48	92
chr1:183854089-183854405	189,364	*Homo sapiens* RPS29 gene for ribosomal protein S29	AB061847	3E-150	99
chr3:36417092-36417971	155,205	Mouse 28S rRNA	X00525	0	91
chrX:51467917-51468014	133,492	*Crocodylus siamensis* 18S rRNA	EU727190	1E-13	93
chr1:89070737-89071827	104,507	*Equus caballus* 28S rRNA	NR_046309	0	99
chrUn:55274673-55275483	98,778	*Homo sapiens* 28S rRNA	M11167	0	91
chrX:87062618-87062767	82,610	*Homo sapiens* 7SL RNA,	NG_002426	2E-54	94
chrUn:64060479-64061987	62,499	*Bubalus bubalis* 18S rRNA	JN412502	0	99
chr20:7063095-7063141	57,594	*Crocodylus porosus* 18S rRNA	EU727191	3E-10	98
chr28:36791911-36792005	56,479	*Homo sapiens* 7SL RNA	M20910	9E-30	94
chr29:1282347-1282467	55,336	*Homo sapiens* 45S pre-rRNA	NR_046235	3E-51	99
chr1:89070491-89070650	53,940	*Equus caballus* hypothetical protein	XM_001916364	1E-60	94

Gene ontology analysis of the sperm transcripts that corresponded to 1,378 annotated equine genes and 3,262 human orthologs produced 10 main functional categories: 1) plasma membrane; 2) mitochondrial ribosomal protein; 3) chemokine receptor and protein folding; 4) transcription regulation; 5) ion binding; 6) cytoskeleton; 7) DNA packaging; 8) chromatin assembly complex; 9) GTPase activator, and 10) RNA processing factors and protein transport. Notably, EST and mRNA sequences with the highest AC values all had known functions in spermatogenesis or sperm-egg interactions (Table 5).

**Table 5:** Structural and functional annotations for mRNAs and ESTs with the highest AC values by RNA-seq.

NCBI Accession No	Gene symbol	Gene name	Location Chr:Mb	AC value	Predicted or known function(s)	Ref.
mRNA						
NM_001081847	*MMP1*	Matrix metallopeptidase 1	7:12.7	11766	spermatogenesis	[94]
NM_001082495	*MMP3*	Matrix metallopeptidase 3	7:12.7	11766	spermatogenesis	[94]
NM_001135102	*TNP2*	Transition protein2	13:33.2	1730	sperm chromatin structure	[95]
NM_001083596	*PRM1*	Protamine 1	13:33.2	1730	sperm chromatin structure	[96]
NM_001159690	*PKM2*	Pyruvate kinase, muscle	1:121.0	297	high fertility sperm	[97]
NM_001163873	*GRP94*	Glucose-regulated protein	28:27.3	238	sperm maturation	[98]
NM_001081764	*COL2A1*	Collagen, type II, alpha 1	6:65.6	222	testes development and descent, male infertility	[98]
NM_001160296	*FBXO9*	F-box protein 9	20:50.6	182	expressed in male germ-cells, sperm differentiation	[95], [99]
NM_001081842	*CASP1*	Caspase 1, apoptosis-related cysteine peptidase	7:14.5	119	male fertility	[100], [101]
NM_001081932	*CRISP2*	Cysteine-rich secretory protein 2	20:47.7	114	sperm capacitation and sperm-egg fusion	[102]
NM_001081874	*CRISP3*	Cysteine-rich secretory protein 3	20:47.7	114	protects sperm from degradation	[103]
dbEST						
CD470129	*NEMF*	Nuclear export mediator factor	1:183.8	242048	sperm-egg interaction	[104]
CX595503	*CTNNBIP1*	Beta-catenin-interacting protein 1	2:41.4	85760	cytoskeletal, cellular morphogenesis, germ cell loss and sterility	[105], [106]
CD466273	*LCP1*	Lymphocyte cytosolic protein 1	7:12.7	11766	sperm maturation	[107]
CD472316	*DNTTIP2*	Deoxynucleotidyltransferase, terminal, interacting protein 2	5:71.1	11420	chromatin remodeling	[108]
CD467145	*FGD3*	FGD1 family, member 3	23:54.9	10551	sperm motility	[109]
CX595998	*LYRM1*	LYR motif containing 4	20:5.6	8565	mitochondrial membrane polarization	[110], [111]
CX596255	*PDIA4*	Protein disulfide-isomerase A4 precursor	4:101.1	4232	spermatogenesis, sperm maturation	[112]
CX592294	*NDUFV2*	NADH dehydrogenase (ubiquinone) flavoprotein 2	8:34.0	1997	expressed in sperm	[113]

Finally, among the 140 sequence tags classified as miRNAs, 82 unique miRNAs were identified of which 17 completely aligned with known equine miRNA genes (Table S7). The majority of miRNAs (76%) showed high expression level (10 AC 100), 13 miRNAs (16%) had AC lower than 10, whereas 6 miRNAs-MIR34B, MIR34C, MIR191, MIR223, MIR1248, and MIR1905C-showed very high expression levels (AC≥100) in stallion sperm (Table S7).

#### Comparison of RNA-seq data with current gene models

Structural annotations of RNA-seq data by ERANGE for pyruvate kinase *(PKM2)*, cysteine-rich secretory protein 3 (*CRISP3)*, protamine 1 (*PRM1)*, and transition protein 2 (*TNP2)* were compared with the current NCBI equine gene models (UCSC Genome Browser; http://genome.ucsc.edu/). The genes were selected due to their known functions in sperm motility, packaging, structure and fertilization (Table 5), and because all four genes were represented by high number of transcripts (AC>100) in the sperm.

**Figure 6:**
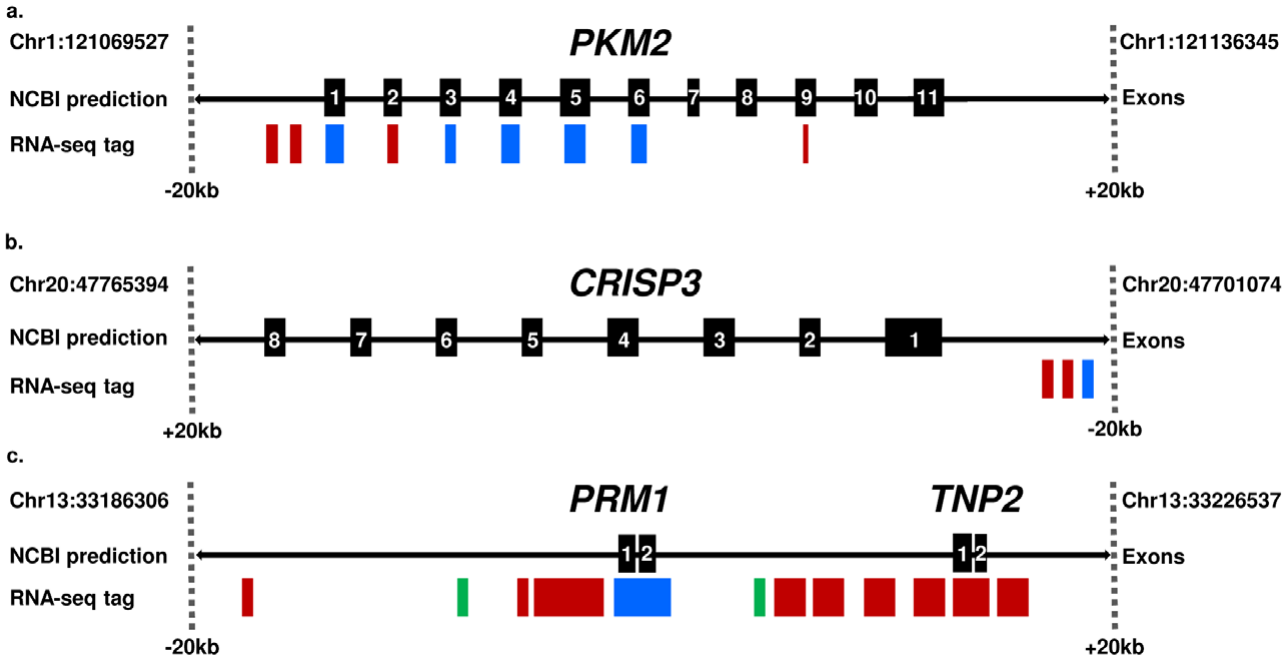
Comparison of RNA-seq data with current equine gene models: (a) *PKM2* showing 9 *in silico* prediction sites, of which two are positioned 5′ upstream to exon 1; (b) *CRISP3* with 3 *in silico* prediction sites, all located 5′ upstream to exon 1; (c) *PRM1* and *TNP2* cluster (the protamine cluster) with 12 *in silico* prediction sites of which only two align with *PRM1* and *TNP2* exons. Black boxes with numbers –exons in current gene models; blue boxes –very highly expressed tags (AC≥100); red boxes–highly expressed tags (10<AC<100); green boxes–tags with medium expression (1≤AC≤100). Exact start and end sites of all mapped tags are presented in Additional file 7.

Of the 9 tags that mapped to *PKM2*, each corresponded to two different NCBI accessions (Table S8) suggesting the presence of two splice variants in stallion sperm. Based on the AC values, the variant comprising of exons 1, 3, 4, 5, and 6 was more abundant than a variant where exons 2 and 9 were included; no tags aligned with exons 7, 8, 10, and 11. However, a relatively abundant (AC = 94.16) sequence tag aligned with a 5′ upstream region of the gene indicating likely presence of an additional exon (Fig. 6a).

The three *CRISP3* sequence tags mapped 5′ upstream from the current gene model and did not align with any of the eight known exons (Fig. 6b). This could indicate inaccurate annotation of the gene in the equine draft assembly (EcuCab2) [42] or the presence of additional 5′exons.

All RNA-seq mapped tags that aligned with *PRM1*, aligned also with *TNP2*, thus having two distinct accession locations (Table S8, Fig. 6c) in this tightly regulated protamine gene cluster [48], [49]. Transcripts with the highest AC values aligned with the two *PRM1* exons, while a number of sequence tags with high to medium AC values mapped 5′ upstream of *PRM1*, and between *PRM1* and *TNP2* (Fig. 6c)-the latter corresponding to parts of the initial joint transcript of the protamine gene cluster [48], [49].

#### Discovery of Y chromosome transcripts in stallion sperm

**Figure 7:**
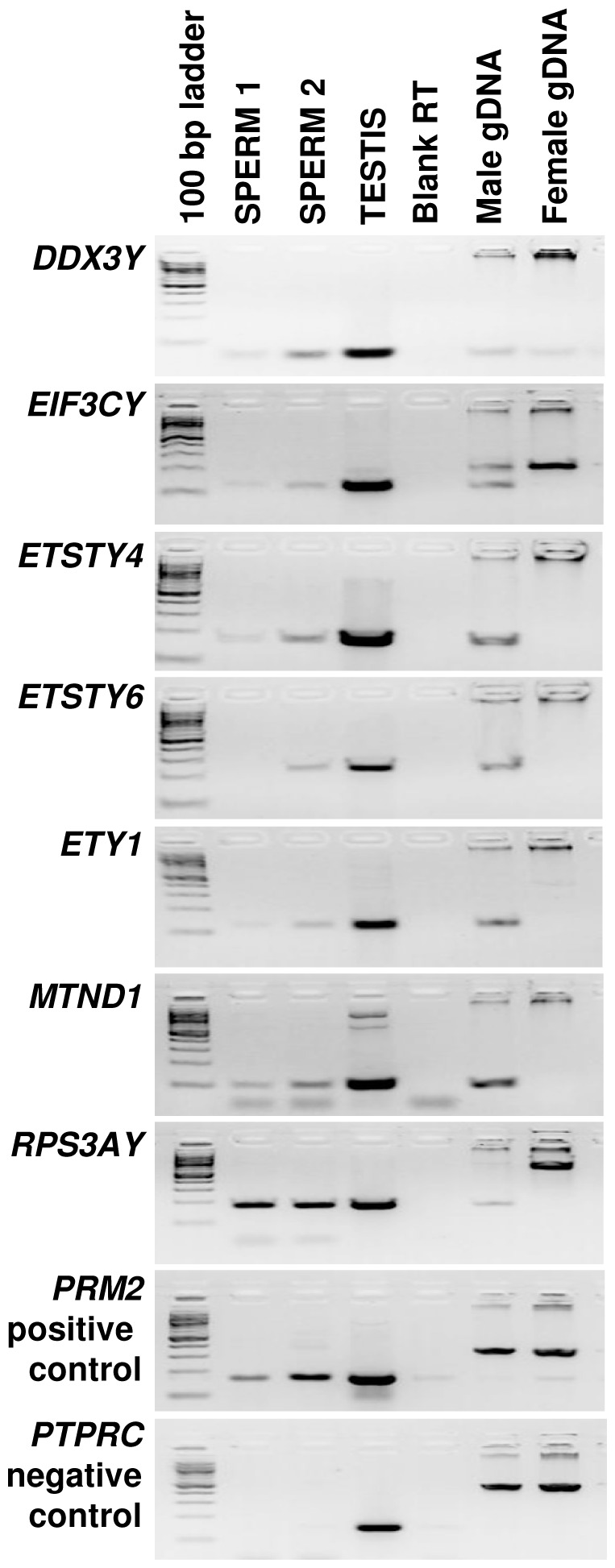
ECAY transcripts in stallion sperm. Agarose gel images showing RT-PCR amplicons of 7 ECAY genes and transcripts in stallion sperm.

Horse Y chromosome sequences are not present in the draft assembly (EcuCab2) [42] or in the whole genome (WG) expression oligoarray [50]. Therefore, we used the recently published catalogue for 29 ECAY genes and ESTs that have cDNA evidence [51], and found seven transcripts in the sperm (Fig. 7). These included one X-degenerate gene (*DDX3Y*), three horse specific novel transcripts (*ETSTY4, ETSTY6* and *ETY1*), and three Y-acquired retrotransposed genes (*EIF3CY, MTND1* and *RPS3AY*).

#### Comparison of microarray and RNA-seq data

The 21,000-element equine gene expression oligoarray is designed to specifically target genes, so that each probe on the array corresponds to a specific gene or EST [50]. The RNA-seq data, on the other hand, comprises the entire transcriptome and multiple sequence tags can be mapped to a genomic region corresponding to one gene. This explains the substantial difference between the number of sperm RNAs detected by microarray analysis (6,761 transcripts) and by RNA-seq (19,257 mapped tags). Nevertheless, the two datasets were similar regarding the number of annotated genes: 3,319 by microarray and 4,504 by RNA-seq. While 65–70% of the microarray transcripts were present among RNA sequences (data not shown), RNA-seq additionally identified miRNAs and over 13,000 anonymous mapped tags. The latter potentially correspond to splice variants of known genes, to genes yet to be annotated, to various non-coding RNAs (ncRNAs), and maybe even to a few novel genes. Importantly, RNA-seq data allowed refined quantitation of RNA transcripts as expressed by the AC values (Table S5), to determine the level of their expression in stallion sperm.

## Discussion

The discovery of haploid transcripts in mammalian sperm dates back to almost two decades when *c-MYC* mRNA [52] and MHC Class I transcripts [53] were detected by RT-PCR in human sperm. Since then, a number of studies have characterized individual transcripts, as well as the global transcriptome of the sperm in normal and subfertile men [6], [8], [28], [31], [54]. In animals, sperm transcripts have been studied in bulls [29], [32], [33], [34], boars [23], [35], and recently in the water buffalo [55] using human microarrays, species-specific small custom-made microarrays, or quantitative PCR. To our best knowledge, the present study is the first global sperm RNA analysis in stallions, though massively parallel sequencing has been recently used to study RNAs in the sperm of men [56] and mice [57]. Our findings that thousands of coding and non-coding RNAs are present in mature stallion sperm are in good agreement with previous research in the field [6], [34], [56], [57].

### Microarray analysis *versus* RNA-seq

Analysis of stallion sperm transcriptome by microarray and RNA-seq in the present study, allowed comparison of the two approaches for the efficiency to detect sperm mRNAs. The information obtained by gene expression microarrays is typically influenced by array design and annotation, with a possible advantage that previously known annotations of array probes will reduce the bioinformatics load of analysis. The 21,351-element equine WG oligoarray [50] used in this study contains 14,531 GO annotated gene products (AgBase: http://www.agbase.msstate.edu/) of which 3,319 were identified in the sperm. In contrast, transcriptional profiling by RNA-seq is unbiased, targets all classes of RNAs, and substantially outperforms microarray in the dynamic range of the expression levels [47], [58]. Indeed, the heterogeneity and expression range of the 19,257 mapped RNA-seq tags in stallion sperm essentially exceeded the microarray data. The downside, however, was limited power of structural annotation of the RNA sequences due to which 70% of mapped tags remained anonymous and will be targets for bioinformatics pipelines in the future. Partial incompatibility between the accession identities of microarray and RNA-seq annotations set additional limitations to efficiently compare the two datasets. We conclude that RNA-seq is certainly the method of choice for global transcriptome analysis and for the discovery of biomarkers for stallion fertility.

### Sperm *versus* testis: selective retention of mRNAs in sperm

The sperm of reproductively normal stallions contained a rich repertoire of about 6,000 mRNAs/ESTs (Figs. 1, 5a) which, according to microarray analysis, represent approximately 50% of the mRNAs found in the testes (Fig. 1), a ratio similar to that reported for men [6]. The ∼11,000 testes transcripts, as determined here by microarray, are close in number to the 12,013 expressed genes recently found in stallion testes by RNA-seq [59].

The majority of mRNA/EST transcripts in stallion sperm were concordant with those in testes (Fig. 1), supporting the prevailing idea that sperm transcripts are solely historical records of spermatogenesis in testes [1], [6], [7], [8], [60], [61]. Therefore, the detection of 60 sperm up-regulated and 165 sperm-enriched transcripts by microarray analysis was a surprise. Because GO analysis of these transcripts showed their direct relevance to sperm functions (Figs. 2, 3; Tables S2, S3), it is tempting to speculate that certain transcriptional products of spermatogenesis are selectively retained in the sperm but not in the testes. This was further supported by GO annotations for the sperm RNAs that corresponded to known genes, mRNAs and ESTs (Table S1, Table 5) showing that the majority of sperm transcripts relate to a few defined functional categories. These included cytoskeleton and G-protein coupled receptor activities, transmembrane transport, ion channels, and mitochondrial ribosomal proteins-functions involved in sperm chemotaxis, capacitation, sperm-egg interactions, and the acrosome reaction [62], [63], [64]. For example, ion channels play an important role in fertilization by facilitating interactions of the sperm with its environment and the egg during capacitation, sperm-egg recognition, and the acrosome reaction [65], [66]. Trans-membrane transport of glycoproteins on the surface of sperm tails, on the other hand, is required for primary binding of the sperm to the zona pellucida during capacitation and sperm-cumulus interaction [67]. Even though the high abundance of olfactory receptors (OR) and the predominance of sensory perception related biological processes in sperm transcriptome (Table S1) seems at first sight bizarre, ORs too are directly involved in sperm functions. There are between 20 and 66 testicular ORs in mammals which play pivotal roles in progesterone activated signal transduction pathways in guiding sperm chemotaxis, capacitation, Ca^2+^- channels and acrosome reaction [64], [68]. The findings also set an important foundation for future research to examine whether the regulation of ORs in individual sperm cells is as tightly controlled as their expression in the central nervous system, where each neuron expresses monoallelically only one particular OR [69]. This might be of value for assisted reproduction and for the improvement of the therapy of subfertility.

### Y chromosome transcripts in stallion sperm

One of the original findings was the detection of seven Y chromosome mRNAs in stallion sperm (Fig. 7). Sequences of the Y chromosome are typically missing from EST and cDNA libraries, from genome sequence draft assemblies, and thus, from expression arrays and gene annotation pipelines. Therefore, Y transcripts in the sperm have been identified only in species with advanced Y chromosome gene catalogues–*DBY*, *SRY*, and *RPS4Y* in men [70] and *Dby* in mice [24]. Given the known role of Y chromosome genes in spermatogenesis and male fertility and their high expression levels in testes [51], [71], the presence of Y transcripts in sperm is not surprising. Although, it is noteworthy that among tens of Y genes expressed in testes, only a few transcripts are retained in the sperm. Among these, *DBY* (alias *DDX3Y*) is of particular interest because it is present in the sperm of all three species–humans, mice and horses. In mice, *Dby* transcripts are retained in the sperm after capacitation and transferred into the oocyte during fertilization. These transcripts are thought to be necessary for the early development because blocking *Dby* with antisense RNA results in inhibition of zygotic development in mice [24]. Given that Y transcripts are delivered to the zygote exclusively by the sperm, the functions of ECAY transcripts in stallion sperm need further investigation.

In summary, functional coherence of the GO categories of stallion sperm coding RNAs is in agreement with the observations in humans that sperm mRNAs are not random untranslated remnants of spermatogenesis but constitute a population of stable full-length transcripts that are selectively retained for functions in fertilization and early development [6], [11].

### Non-coding RNAs: rRNAs, miRNAs and lncRNAs

Direct sequencing of stallion sperm total RNA allowed the discovery and identification of RNA species other than mRNAs. Among these, ribosomal RNAs (rRNAs) comprised a substantial portion of mapped tags with very high AC values (Table 4). This was a surprise because it has been a common knowledge that the sperm are depleted of rRNA [6], [72]. Absence of intact 18S and 28S rRNA peaks has been shown in most microarray-based sperm transcriptome studies [6], [32], [35], [38] and is an established standard for sperm RNA quality evaluation [73]. Recent RNA-seq of human sperm transcriptome [56] reveals that the truth lies in the middle: 18S and 28S are the most abundant (80%) sperm transcripts but they are not intact. Sperm rRNAs undergo selective cleavage which specifically destroys full-length rRNAs but does not affect mRNAs or small non-coding RNAs. Cleavage of sperm rRNAs is needed to ensure translational cessation and prevent spurious protein synthesis in the sperm. These findings explain the presence of rRNAs in the stallion sperm in this study but also clarify why 18S and 28S peaks are absent from sperm RNA quality control electropherograms [73].

One of the most exciting results was the discovery of 82 sperm miRNAs (Table S7) which comprised 0.73% of all RNA-seq mapped tags (Fig. 4) and were annotated according to the *in silico* detection of miRNAs in the horse genome [74]. The number of miRNAs in stallion sperm was comparable with the 68 miRNAs found in human spermatozoa [13], and several stallion miRNAs were the same as identified in the sperm of men [75], boars [76] and mice [57], [77]. Among the latter, the most noteworthy were the sperm-borne miRNAs which are required for the first cleavage division and are found in mouse sperm and one-cell embryos but not in the oocytes or embryos past the one-cell stage. Three such miRNAs [77], MIR34B, MIR34C and MIR449A, were highly abundant (AC≥100) in stallion sperm (Table S7). While the functions of sperm microRNAs in equine biology are yet to be determined, recent discoveries in mouse and humans suggest that sperm miRNAs, as well as novel piRNA- and tRNA-derived small RNAs [57], [78], regulate gene expression in the early zygote either by direct interaction with mRNA or via epigenetic mechanisms [13], [20], [21], [77], [79]. For example, miR-124, also found in stallions, is critical for the establishment of a distinct, heritable chromatin structure in the promoter region of *Sox9* and is responsible for RNA-mediated epigenetic control of embryonic and adult growth in mice [79]. Further, recent comparative study on birth and expression evolution of mammalian miRNA genes [80] indicates the particular importance of X-linked miRNAs in testes where they are potentially involved in diverse functions during spermatogenesis. These X derived miRNAs tend to be duplicated and have higher expression levels than autosomal miRNAs. Though the functions of miRNAs in testes and sperm are likely different, it is worth mentioning that among the 82 sperm miRNAs identified in this study, six were derived from the X chromosome of which MIR223 has high expression level (AC = 295; Table S7).

The discovery of over 100 sperm miRNA sequence tags of which 82 could be aligned with unique miRNAs evidenced that to some extent the small RNA fraction can be successfully targeted by global transcriptome sequencing, without special small RNA library construction. However, given that mammalian species on average have about 300 miRNAs [80] and that sperm are enriched with mse-tsRNAs (mature sperm-enriched tRNA-derived small RNAs) [78] and other small non-coding RNAs [57] with likely functions in development, additional studies are needed for in depth analysis of the small non-coding RNA fraction of stallion sperm.Male germ cells also contain transcripts of long non-coding (lnc) regulatory RNAs [21] which are longer than 200 nucleotides, have little or no protein-coding capacity, and regulate gene expression through a diversity of mechanisms [81]. Because only three lncRNA genes are available for the horse in the Long Non-Coding RNA Database (http://lncrnadb.com/) and lncRNA sequences are not conserved across species [81], the RNA-seq annotation pipelines (ERANGE, AgBase) [47] did not identify any lncRNAs. However, we anticipate that among the over 13,000 RNA-seq tags that could not be annotated in this study, many represent small and long regulatory RNA species.

### Functions of sperm transcripts

Recent studies have essentially challenged the prevalent concept that the sperm are transcriptionally and translationally dormant cells [82] and that sperm transcripts have no functions of their own [1], [6], [7], [8], [11], [61], [83]. For example, there is evidence that the mature sperm possess an efficient RNA polymerase machinery for transcription, mRNA splicing and for reverse transcription of the primary RNA into stable cDNAs [84], majority of which are delivered during fertilization to the zygote [16]. Sperm mRNAs can be *de novo* translated using mitochondrial-type ribosomes and at least 26 such sperm-translated proteins are known to be required during capacitation, sperm-egg interactions and fertilization [17], [18], [19], [85]. Also, sperm coding and non-coding RNAs are thought to have a role in stabilizing sperm chromatin and facilitating the selective escape of sequences necessary for early development from repackaging by protamines [15]. This is in line with our findings that stallion sperm mRNAs are not retained randomly but form a distinct population with functions directly relevant to sperm-egg interactions, fertilization and embryonic development. Furthermore, the presence of non-coding regulatory RNAs suggests that like in mice, RNAs can serve as epigenetic modifiers of gene regulation in early equine development [10], [20], [21], [86]. Despite these recent advances, functions of the majority of RNAs found in mammalian sperm remain to be identified [10] and need further investigation.

The primary practical goal of sperm transcriptome analysis in humans and animals is the detection of transcripts that could serve as biomarkers or diagnostic tools for fertility evaluation. For example, elevated protamine mRNA retention in human sperm is an indication of abnormal protamine translation and infertility [27]. Also, consistent and biologically relevant differences in sperm mRNA expression profiles have been found between fertile men and men with teratozoospermia [26], cryptorchidism [28] and idiopathic infertility [30], [31]. In bulls, DE sperm transcripts have been associated with high or low sperm motility [29], as well as with overall high- and low-fertility [34]. In boars, statistically significant differences in sperm mRNA profiles have been associated with seasonal changes in the reproductive status [36]. Overall, the current knowledge about sperm transcriptome in men and animals suggests that sperm RNA profiles could be used as a genetic fingerprint of normal fertile males and as a molecular diagnostic platform for male infertility. In this respect, the results of the present study, particularly the expression data for sperm miRNAs and the mRNAs relevant to sperm functions, set a foundation for the development of sperm-based markers for fertility evaluation in stallions in the future.

### RNA-seq data and structural annotation of the horse genome

While the primary goal of this study was to characterize in detail the transcriptome of stallion sperm, the generated RNA-seq data is a valuable resource for the improvement of horse genome structural annotation [54]. This was illustrated by suggesting additional exons, splice variants or another genomic location for four important sperm genes-*PKM2, CRISP3, TNP2* and *PRM1* (Fig. 6). Thus, the RNA-seq data is a valuable resource to improve the structural annotation of the horse genome, and for the discovery of novel genes and regulatory RNAs.

## Methods

### Ethics statement

Procurement of stallion semen and testes was performed according to the *United States Government Principles for the Utilization and Care of Vertebrate Animals Used in Testing, Research and Training* and were approved by the Clinical Research Review Committee (CRRCs #08–19; #08–33; #09–32; #09–47) and Animal Use Protocol #2009–115 at Texas A&M University, supplemented with Informed Owner Consent From stating that owners of the stallions gave permission for their animals to be used in this study.

### Samples

Fresh ejaculates from five reproductively normal stallions were collected using an artificial vagina (Missouri model). The ejaculates were first evaluated for sperm concentration, motility characteristics and morphological features [87], [88], followed by purification from somatic cells and immature sperm by EquiPure™ (Nidacon International, Sweden) discontinuous gradient centrifugation [73]. Testes samples were obtained from four normal stallions by castration. Purified sperm and testes were stored in RNAlater (Ambion) at −80°C until use.

### RNA isolation and evaluation

Total RNA was isolated from sperm with TRIzol reagent (Invitrogen) as described by Das and colleagues [73], and from testes using RNeasy mini elute kit (Qiagen) and manufacturer's protocol. The RNA samples were cleaned from genomic DNA (gDNA) with Turbo DNase kit (Applied Biosystems/Ambion) and purified with RNeasy MinElute Cleanup kit (Qiagen). The quantity and quality of isolated RNA were evaluated with spectrophotometer (NanoDrop 1000, Thermo Fisher Scientific), Bioanalyzer (Agilent Technologies), and reverse transcriptase PCR (RT-PCR) using primers for sperm- and testes-specific *PRM2* (protamine 2), and somatic cell-specific *PTPRC* (protein tyrosine phosphatase, receptor type, C) (Fig. S2) [73]. The spectrophotometer OD values for all total RNA samples must to be 1.70–1.75 for absorbance ratios A260/A280, indicating that the RNA is free from proteins and organic compounds [89]. The Bioanalyzer profiles distinguish between testes and sperm total RNA: RNA Integrity Number (RIN) above 8 and two peaks corresponding to 18S and 28S rRNAs are indicators for the good quality of testes RNA; in contrast, sperm is depleted of intact rRNA (Fig. S2) [73]. RT-PCR with intron-spanning primers for *PRM2* validate that all RNA samples are free of genomic DNA, while no amplification of *PTPRC* in sperm indicates that the sperm RNA is not contaminated with RNA from somatic cells (Fig. S2) [73].

### RNA linear amplification

For microarray hybridizations, sperm and testes total RNA was subjected for two rounds of linear amplification by T3/T7 promoter synthesis with RNA Amplification RampUp kit (Genisphere) following manufacturer's instructions. Starting with 20–30 ng of total RNA, about 20–60 µg of sense-strand mRNA was obtained and stored at −80°C until use.

### Expression microarray hybridizations

Four different testes and five sperm RNA samples were used for microarray hybridizations. Testes samples were pooled to generate a reference RNA for normal stallion testes, while sperm samples were used individually. Individual sperm and pooled testes RNA was converted into cDNA and labeled with Cy3 or Cy5 using 3DNA Array 900MPX Detection kit (Genisphere). Transcriptional profiles of stallion sperm and testes were studied by hybridization to the Texas A&M 21,351-element equine WG expression oligoarray [50]. Each hybridization experiment comprised a pair of differently labeled (Cy3 or Cy5) RNAs: the testes reference and one of the five sperm samples. Including a dye swap, a total of ten microarray hybridizations were conducted in a Sure Hyb hybridization chamber (Agilent Technologies) overnight, followed by post-hybridizaton washes in pre-warmed (42°C) 2× SSC with 0.2% SDS and 0.2× SSC at room temperature for 15 min each.

### Microarray data analysis

The slides were scanned with a Gene Pix 4100B scanner at 5 micron resolution (Molecular Devices). Spot-finding and quantification of array images was carried out using *Gene Pix Pro 6.1* software and the data were stored as GenePix Results (.gpr) files. The raw intensity data were normalized within individual arrays using print-tip LOWESS method [90]. To be considered significant, the signal for a candidate had to be above a threshold value (SNR ≥2) determined according to the fluorescence output of the negative controls printed on the microarray. Bayesian t-test was performed to consider DE genes between the sperm and testes: signal FC >2 and *p* value 0.05 were considered significant. The normalized data were analyzed with Bioconductor LIMMA package in the *R* computing environment, followed by GO analysis using DAVID Bioinformatics Resources (http://david.abcc.ncifcrf.gov/) to describe those molecular functions and biological processes that appeared to be influential.

### Validation by quantitative real-time PCR (qRT-PCR)

The cDNA was synthesized from 2 µg of linearly amplified testes and sperm RNA with SuperScript VILO cDNA synthesis kit (Invitrogen), purified with MinElute PCR purification kit (Qiagen), and evaluated for quantity and quality with a NanoDrop spectrophotometer (Thermo Fisher Scientific). Aliquots of cDNA were stored at −20°C until use. Exon spanning primers for qRT-PCR were designed for selected genes (Table 1) using Primer3 *ver* 0.4.0 software [91], and the efficiency of all primers was evaluated by making a standard curve in the sperm and testes samples. Duplicate qRT-PCR reactions in triplicate experiments were carried out on a Light Cycler® 480 (Roche Diagnostics) along with two housekeeping genes (*ACTB*, β-actin and *PPIA*, peptidylprolyl isomerase α) as controls. Each qRT-PCR assay used ∼100 ng of mRNA in a 20 µL reaction with 1× Universal SYBR® Green Master Mix (Applied Biosytems, CA) and 300 nM primers. The results were analyzed with LightCycler 480 Software v1.5 by calculating log_2_^−ΔΔCt^; the *P*-value was calculated by performing student's t-test and *p*<0.05 was considered significant. Scatter plots for qRT-PCR statistics were generated in Microsoft Excel (Fig. S1).

### Detection of Y chromosome transcripts in stallion sperm

Reverse transcriptase PCR experiments on stallion sperm and testes were carried out according to standard protocol [73] using primers for 29 known horse Y chromosome genes and transcripts with cDNA evidence [51], along with primers for *PRM2* and *PTPRC* as positive and negative controls, respectively.

### RNA-seq library construction and sperm RNA sequencing

Total RNA from the sperm of two reproductively normal stallions was used for next generation sequencing (NGS) on the ABI SOLiD 4 platform at Cofactor Genomics (ST. Louis, MO, USA).-Total RNA (500 ng) was directly used for SOLiD single-end RNA sequencing fragment library construction according to the ABI protocol (http://www.cofactorgenomics.com/faq) [92]. First strand cDNA was directly generated from total RNA using 4 µL of random hexamers (ABI) and SuperScript II Reverse Transcription Kit (Invitrogen) in a 30 µL final volume, following the manufacturer's instructions. The second strand cDNA was generated using 10 µL of 5× second strand buffer (500 mM Tris-HCl, pH 7.8; 50 mM MgCl2; 10 mM DTT), 30 nmol dNTPs; 2 U of RNase H, and 50 U of DNA Pol I (Invitrogen), and incubated at 16 °C for 2.5 h. The double-stranded DNA (dsDNA) was purified with QIAquick PCR purification kit (Qiagen) and the concentration was quantified. From each sample, ∼100–200 ng of cDNA was fragmented using Covaris S2 System (Covaris, Inc.). Sequencing libraries were generated with SOLiD Fragment Library Construction Kit (ABI) as described elsewhere [92]. Briefly, fragmented cDNA was end-repaired with Polishing Enzyme 1 and End Polishing Enzyme 2 (ABI); adapter ligated with SOLiD P1 and P2 adaptors, size selected for 200 to 230 bp on a SOLiD Library Size Selection gel, followed by nick translation and PCR amplification using Library PCR Primers 1 and 2 and Platinum PCR Amplification Mix. Amplified libraries were column purified, quantified using the SOLiD Library TaqMan Quantitation Kit, and applied on ABI SOLiD sequencer at a concentration of 10 ng per lane.

### RNA sequence analysis and annotation

The 50 bp single-end SOLiD raw reads were directly aligned with the horse reference sequence EcuCab2 [42] using ABI aligner software (NovoalignCS version 1.00.09, http://www.novocraft.com/) which uses multiple indexes in the reference genome, identifies candidate alignment locations for each primary read, and scores alignment locations using the Needleman-Wunsch algorithm [93]. The alignment parameters allowed the minimum number of 30 good quality bases for a read (l = 30); the highest alignment score acceptable for the best alignment was 140 (t = 140), whereas a default threshold was calculated from read length and genome size such that an alignment to a non-repeat should have a quality higher than 30; the number of alignments recorded for a read during the iterative search process, i.e., the number of alignments with score equal to the best alignment was 10 (e = 10). If a read was unaligned, it was shortened by 1 base and tried again. Alignments in repetitive sequences were discarded by removing reads with multiple similarly scoring alignments. The single highest-scoring alignment for each raw read was mapped. Sequence alignment and alignment clustering to define expressed loci and perform linear normalization across the two sperm RNA samples was carried out with a software package EXpression analysis Pipeline, EXP (Cofactor Genomics).

*Gene expression* level or *average coverage* (AC) was calculated by normalizing each sample to the fewest reads and directly comparing different loci. Expression level of a transcript was estimated from the number of reads that mapped to that transcript. The variability present in sequencing depths in different samples was taken care of by the use of two biological replicates. Sequencing depth at each locus and differences in gene expression (AC) between the two sperm samples were calculated using log (base_2_) ratio, thus showing the association between the two samples. Some differences in alignment of data in biological replicates were likely due to sequencing errors and chance alignments which is a significant problem for short reads and low alignment scores [46]. To combat the high false-positive rate, we focused on a high-quality subset of the data consisting of sequence variants supported by different independent reads. Sequencing reads were computationally categorized according to their AC and chromosomal location. This categorization was conducted comparatively with respect to a present horse genome draft sequence assembly, and normalized count of the number of mapped position was calculated. This count served as a proxy for the transcripts with true abundance in the sample. Expression directories were divided into four categories according to the sum of AC values: very high–AC≥100; high −10<AC<100; medium −1≤AC≤10, and low AC<1. Loci with low expression in both sperm samples were removed from further analysis because they represented the least compelling evidence of expression. Genomic locations of all mapped transcripts were retrieved using Python VS 2.66 script.

Structural annotation of genes for all sequence tags with AC≥1 was conducted in two categories: i) a homology-based approach with the human genome (AgBase GOanna; http://agbase.msstate. edu/cgi-bin/tools/GOanna.cgi) and ii) direct annotation with the horse genome using the Enhanced Read Analysis of Gene Expression (ERANGE) with a ±20 kb window for recognized chromosomal locations. Matches were categorized as: F–falling within gene boundaries; P–partially falling within gene boundaries, and A–adjacent, falling into extended gene boundaries within the expanded ERANGE window. Annotated genes were functionally analyzed and clustered for GO terms in DAVID Bioinformatics Resources (http://david.abcc.ncifcrf.gov/) with medium classification stringency for all parameters.

## Supporting Information

Figure S1 **Scatter plots of qRT-PCR statistics for DE genes in sperm and testes by microarray analysis (see also** **Fig. 3** **).** **A** Sperm up-regulated genes: a. *PAD16*, *p*-value 0.025063716, Fold change -17.74531191(sperm), 5.757703597 (testis); b. *DNAJC16B*, *p*-value 0.000370874, Fold change -59.55886046 (sperm), 1.329895004 (testis); c. *DCDC2*, *p*-value 0.009505038, Fold change -41.79889717 (sperm), 1.054235336 (testis); d. *CTTN*, *p*-value 0.025377064, Fold change -114.2727567 (sperm), 17.61968043 (testis); e. *REEP6*, *p*-value 5.65337E-05, Fold change -858.1806418 (sperm), 5.757703597 (testis); f. *ARID5B*, *p*-value 0.029703844, Fold change -2.675065645 (sperm), 0.370649473 (testis); g. *ATG12*, *p*-value 0.079897582, Fold change -4.477059424 (sperm), 0.693040106 (testes); **B** Sperm down-regulated genes: a. *GSTA1*, *p*-value 0.008611828, Fold change -0.427023 (sperm), 8.3 (testes); b. *DYNTL1*, *p*-value 0.028173, Fold change -0.777409 (sperm), 2.95 (testes); c. *SPA17*, *p*-value 0.016193, Fold change -0.569896 (sperm), 1.75 (testes); d. *CTTN*, *p*-value 3.3E-05, Fold change -0.24142 (sperm), 4.14.
(TIF)

Figure S2 **Sperm RNA quality check.** **A** Bioanalyzer analysis showing that mature sperm is devoid of intact ribosomal 18S and 28S RNA; **B** RT-PCR with sperm and testis specific *PRM2* (left) and sperm-negative *PTPRC*.
(TIF)

Table S1 **Gene Ontology classifications and terms for 3,319 sperm transcripts by microarray analysis**. This table contains GO analysis statistics for all annotated genes that were expressed in sperm by microarray analysis. The GO categories i) Biological process, ii) Molecular function, and iii) Cellular component are shown on separate spreadsheets; **Count**-number of genes associated with this gene set; **Percentage**-genes associated with this gene set/total number of query genes; ***P*** **-value**-modified Fisher Exact *P*-value; **Genes**-the list of genes from query set that are annotated to this gene set.
(XLS)

Table S2 **Most significant (p<0.001) GO terms for sperm transcripts identified by microarray analysis (count-number of genes associated with this gene set).**
(DOCX)

Table S3 **Gene Ontology classifications and terms for 165 sperm-enriched transcripts by microarray analysis.** This table lists GO analysis statistics for the sperm-enriched genes. The GO categories i) Biological process, ii) Molecular function, and iii) Cellular component are shown on separate spreadsheets; **Count**-number of genes associated with this gene set; **Percentage**-genes associated with this gene set/total number of query genes; ***P*** **-value**-modified Fisher Exact *P*-value; **Genes**-the list of genes from query set that are annotated to this gene set.
(XLSX)

Table S4 **Differentially expressed genes (n = 155) between the sperm and the testes.** A list of the 60 sperm up-regulated and 95 sperm down-regulated genes, their NCBI and RefSeq accession numbers, logFC-log2 fold change in expression between sperm and testes; AveExpr-average log2-expression level of that gene across red-green channels, and *P*-value; NULL–no annotation; #NA = unknown.
(XLS)

Table S5 **Mapped RNA sequence tags (n = 19,257) from the sperm of the two stallions.** The table presents the following information for each mapped sequence tag: i) genomic location, ii) average coverage in sperm 1 (AC1) and sperm 2 (AC2), and iii) log2 ratios between AC1 and AC2. Columns at the left are sorted by AC1 and columns at the right by AC2. Mapped tags with AC≥1 are shaded grey.
(XLS)

Table S6 **List of the 136 genes from RNA-seq data with structural annotations both in the horse and the human genome.**
(DOCX)

Table S7 **The 82 sperm micro RNAs discovered by RNA-Seq.**
(DOCX)

Table S8 **Correspondence of the RNA-Seq data with the current NCBI gene models for** ***PKM2*** **,** ***CRISP3*** **,** ***TNP2*** **and** ***PRM1*** **.**
(DOCX)

Table S9 **Alignment and coverage statistics for RNA-seq reads in the horse genome.**
(XLSX)
