# Supplementary figures and images for: Stallion Sperm Transcriptome Comprises Functionally Coherent Coding and Regulatory RNAs as Revealed by Microarray Analysis and RNA-seq
Source: PLoS One. 2013 Feb 11;8(2):e56535. doi: 10.1371/journal.pone.0056535 (PMC3569414; doi:10.1371/journal.pone.0056535)

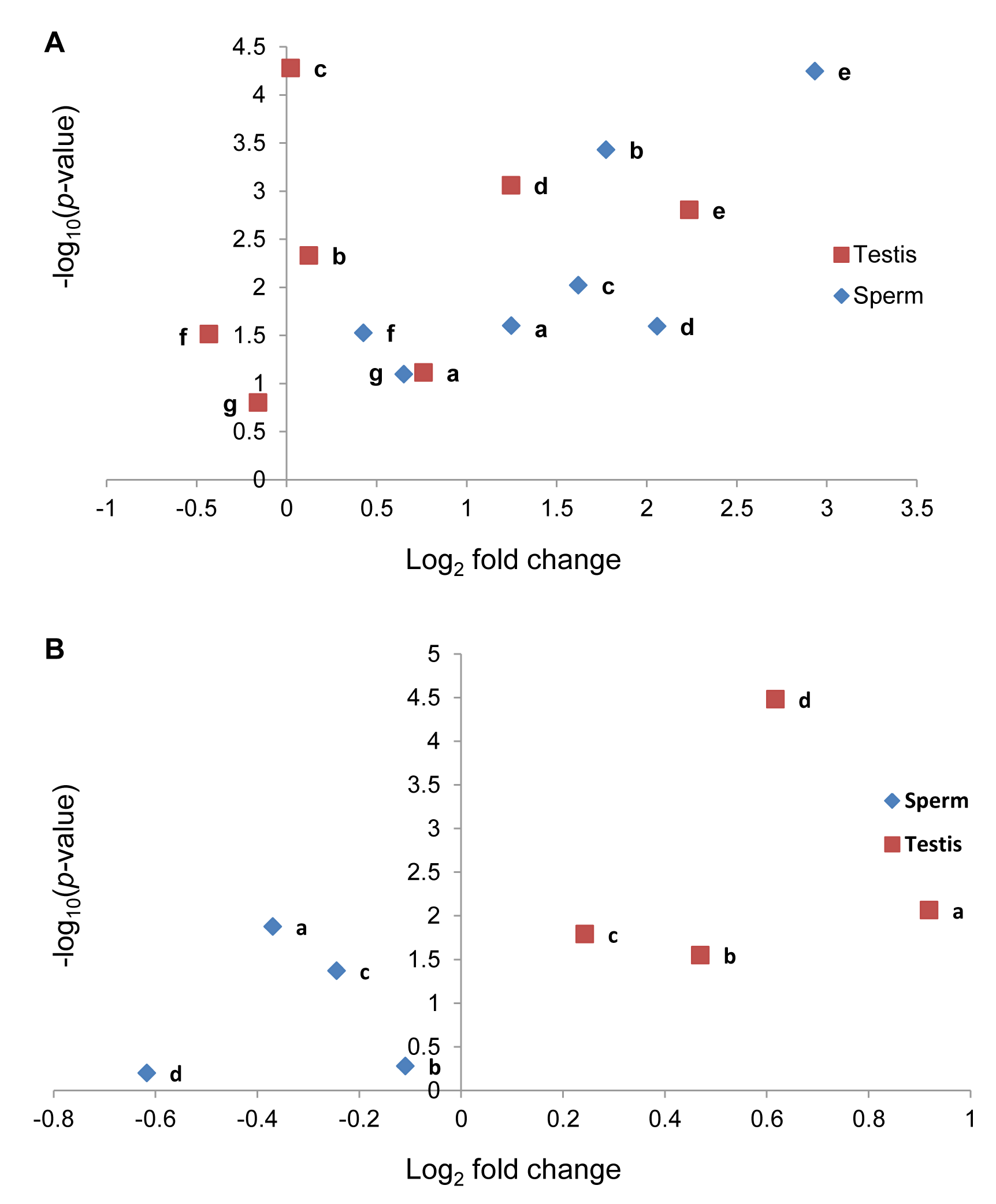

Supplement: Figure S1 — Scatter plots of qRT-PCR statistics for DE genes in sperm and testes by microarray analysis (see also Fig. 3 ). A Sperm up-regulated genes: a. PAD16, p-value 0.025063716, Fold change -17.74531191(sperm), 5.757703597 (testis); b. DNAJC16B, p-value 0.000370874, Fold change -59.55886046 (sperm), 1.329895004 (testis); c. DCDC2, p-value 0.009505038, Fold change -41.79889717 (sperm), 1.054235336 (testis); d. CTTN, p-value 0.025377064, Fold change -114.2727567 (sperm), 17.61968043 (testis); e. REEP6, p-value 5.65337E-05, Fold change -858.1806418 (sperm), 5.757703597 (testis); f. ARID5B, p-value 0.029703844, Fold change -2.675065645 (sperm), 0.370649473 (testis); g. ATG12, p-value 0.079897582, Fold change -4.477059424 (sperm), 0.693040106 (testes); B Sperm down-regulated genes: a. GSTA1, p-value 0.008611828, Fold change -0.427023 (sperm), 8.3 (testes); b. DYNTL1, p-value 0.028173, Fold change -0.777409 (sperm), 2.95 (testes); c. SPA17, p-value 0.016193, Fold change -0.569896 (sperm), 1.75 (testes); d. CTTN, p-value 3.3E-05, Fold change -0.24142 (sperm), 4.14. (TIF) [file pone.0056535.s001.tif]

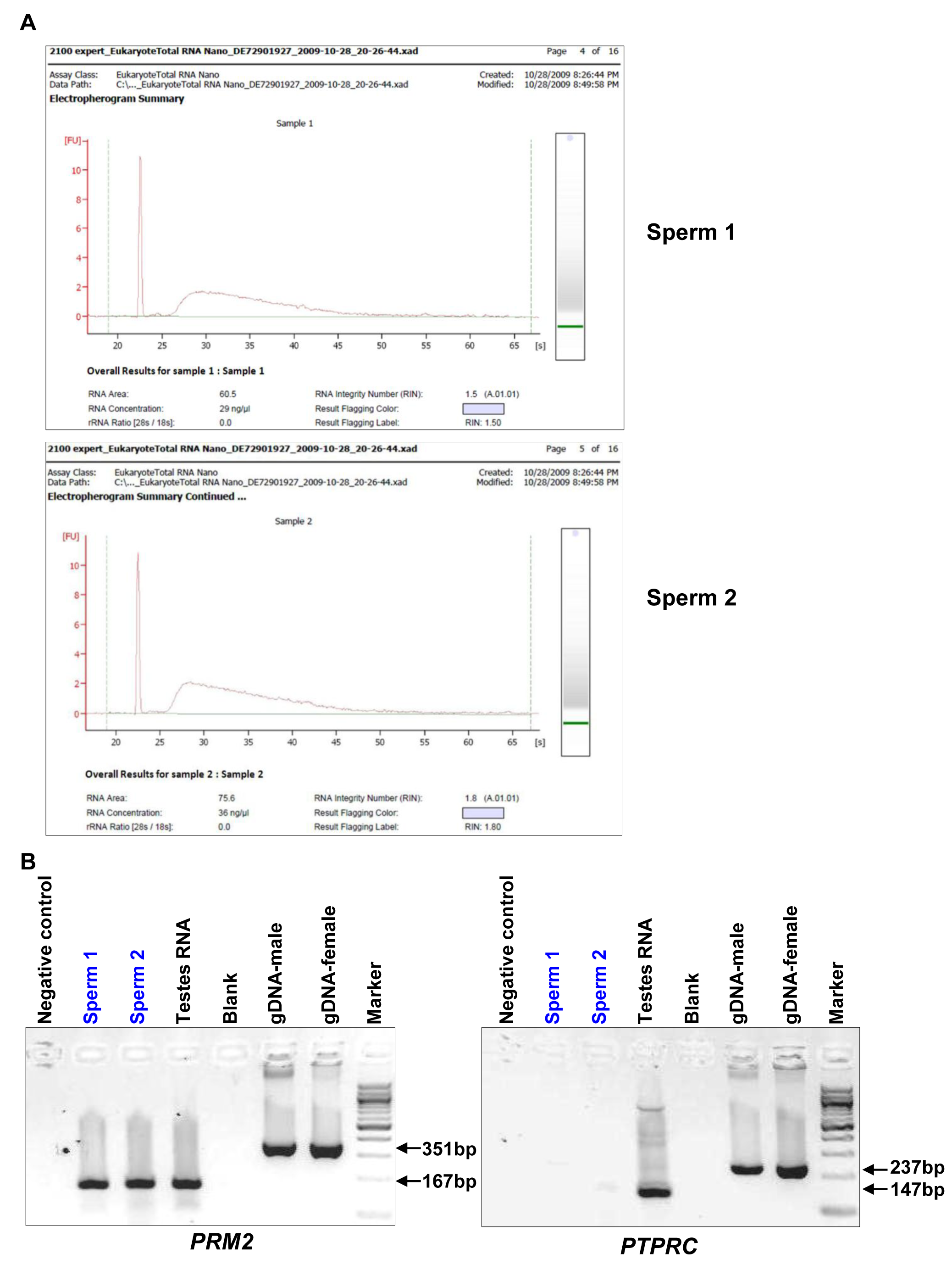

Supplement: Figure S2 — Sperm RNA quality check. A Bioanalyzer analysis showing that mature sperm is devoid of intact ribosomal 18S and 28S RNA; B RT-PCR with sperm and testis specific PRM2 (left) and sperm-negative PTPRC. (TIF) [file pone.0056535.s002.tif]
