# Supplementary material for: Stallion Sperm Transcriptome Comprises Functionally Coherent Coding and Regulatory RNAs as Revealed by Microarray Analysis and RNA-seq
Source: PLoS One. 2013 Feb 11;8(2):e56535. doi: 10.1371/journal.pone.0056535 (PMC3569414; doi:10.1371/journal.pone.0056535)
Supplement: Table S2 — Most significant (p<0.001) GO terms for sperm transcripts identified by microarray analysis (count-number of genes associated with this gene set). (DOCX) [file pone.0056535.s004.docx]

**Table S2: Most significant (p<0.001) GO terms for sperm transcripts identified by microarray analysis (**count - number of genes associated with this gene set).

| **A. BIOLOGICAL PROCESS** | **Count** | ***P-*Value** |
| --- | --- | --- |
| GO:0007608~sensory perception of smell | 218 | 3.61E-65 |
| GO:0007606~sensory perception of chemical stimulus | 227 | 1.82E-61 |
| GO:0007600~sensory perception | 273 | 1.13E-38 |
| GO:0007186~G-protein coupled receptor protein signaling pathway | 340 | 2.04E-37 |
| GO:0050890~cognition | 285 | 5E-34 |
| GO:0050877~neurological system process | 326 | 1.47E-25 |
| GO:0007166~cell surface receptor linked signal transduction | 433 | 2.28E-20 |
| GO:0055085~transmembrane transport | 147 | 4.1E-10 |
| GO:0006812~cation transport | 134 | 1.68E-07 |
| GO:0006811~ion transport | 173 | 4.59E-07 |
| GO:0030001~metal ion transport | 105 | 9.42E-05 |
| GO:0006367~transcription initiation from RNA polymerase II promoter | 24 | 0.000186 |
| GO:0046942~carboxylic acid transport | 41 | 0.000268 |
| GO:0015849~organic acid transport | 41 | 0.000312 |
| GO:0006354~RNA elongation | 19 | 0.000505 |
| GO:0006890~retrograde vesicle-mediated transport, Golgi to ER | 11 | 0.000617 |
| GO:0006368~RNA elongation from RNA polymerase II promoter | 18 | 0.000686 |
| GO:0045944~positive regulation of transcription from RNA polymerase II promoter | 83 | 0.000731 |
| GO:0006352~transcription initiation | 26 | 0.000741 |
| GO:0008643~carbohydrate transport | 21 | 0.000752 |
| GO:0006814~sodium ion transport | 36 | 0.000775 |
| GO:0005513~detection of calcium ion | 7 | 0.000836 |
| GO:0048199~vesicle targeting, to, from or within Golgi | 8 | 0.000917 |
| **B. MOLECULAR FUNCTION** |  |  |
| GO:0008270~zinc ion binding | 480 | 4.73E-46 |
| GO:0046914~transition metal ion binding | 502 | 1.32E-30 |
| GO:0003677~DNA binding | 419 | 2.16E-24 |
| GO:0046872~metal ion binding | 637 | 3.41E-20 |
| GO:0043169~cation binding | 639 | 1.44E-19 |
| GO:0005524~ATP binding | 279 | 1.26E-18 |
| GO:0032559~adenyl ribonucleotide binding | 281 | 2.24E-18 |
| GO:0043167~ion binding | 639 | 6.19E-18 |
| GO:0030554~adenyl nucleotide binding | 287 | 1.8E-16 |
| GO:0001883~purine nucleoside binding | 287 | 1.2E-15 |
| GO:0001882~nucleoside binding | 288 | 1.63E-15 |
| GO:0030528~transcription regulator activity | 258 | 1.49E-11 |
| GO:0000166~nucleotide binding | 352 | 9.96E-11 |
| GO:0003779~actin binding | 79 | 1.84E-10 |
| GO:0032553~ribonucleotide binding | 296 | 2.61E-10 |
| GO:0032555~purine ribonucleotide binding | 296 | 2.61E-10 |
| GO:0017076~purine nucleotide binding | 302 | 2.26E-09 |
| GO:0004674~protein serine/threonine kinase activity | 92 | 5.09E-09 |
| GO:0003723~RNA binding | 134 | 1.4E-08 |
| GO:0003712~transcription cofactor activity | 79 | 3.31E-08 |
| GO:0003774~motor activity | 41 | 5E-08 |
| GO:0008134~transcription factor binding | 101 | 7.51E-08 |
| GO:0030695~GTPase regulator activity | 84 | 9.77E-08 |
| GO:0016563~transcription activator activity | 84 | 1.89E-07 |
| GO:0042623~ATPase activity, coupled | 62 | 2.17E-07 |
| GO:0004672~protein kinase activity | 113 | 2.28E-07 |
| GO:0060589~nucleoside-triphosphatase regulator activity | 84 | 2.62E-07 |
| GO:0008092~cytoskeletal protein binding | 97 | 4.22E-07 |
| GO:0016887~ATPase activity | 71 | 4.55E-07 |
| GO:0004386~helicase activity | 37 | 2.52E-06 |
| GO:0005083~small GTPase regulator activity | 59 | 3.1E-06 |
| GO:0003777~microtubule motor activity | 25 | 3.38E-06 |
| GO:0070035~purine NTP-dependent helicase activity | 29 | 3.65E-06 |
| GO:0008026~ATP-dependent helicase activity | 29 | 3.65E-06 |
| GO:0005198~structural molecule activity | 112 | 4.07E-06 |
| GO:0003713~transcription coactivator activity | 49 | 4.15E-06 |
| GO:0003735~structural constituent of ribosome | 41 | 5.68E-06 |
| GO:0005201~extracellular matrix structural constituent | 26 | 8.56E-06 |
| GO:0003700~transcription factor activity | 155 | 2.39E-05 |
| GO:0003682~chromatin binding | 36 | 3.38E-05 |
| GO:0005089~Rho guanyl-nucleotide exchange factor activity | 22 | 6.52E-05 |
| GO:0015247~aminophospholipid transporter activity | 9 | 9.57E-05 |
| GO:0004012~phospholipid-translocating ATPase activity | 9 | 9.57E-05 |
| GO:0005085~guanyl-nucleotide exchange factor activity | 34 | 0.000242 |
| GO:0005088~Ras guanyl-nucleotide exchange factor activity | 23 | 0.000335 |
| GO:0016564~transcription repressor activity | 58 | 0.000408 |
| GO:0008135~translation factor activity, nucleic acid binding | 24 | 0.000654 |
| **CELLULAR COMPONENT** |  |  |
| GO:0016021~integral to membrane | 1261 | 9.26E-51 |
| GO:0031224~intrinsic to membrane | 1283 | 3.7E-47 |
| GO:0005886~plasma membrane | 810 | 2.49E-12 |
| GO:0005887~integral to plasma membrane | 272 | 1.89E-06 |
| GO:0031226~intrinsic to plasma membrane | 273 | 8.58E-06 |
| GO:0030663~COPI coated vesicle membrane | 10 | 2.26E-05 |
| GO:0030126~COPI vesicle coat | 9 | 9.65E-05 |
| GO:0030137~COPI-coated vesicle | 10 | 0.000307 |
