# Supplementary material for: Stallion Sperm Transcriptome Comprises Functionally Coherent Coding and Regulatory RNAs as Revealed by Microarray Analysis and RNA-seq
Source: PLoS One. 2013 Feb 11;8(2):e56535. doi: 10.1371/journal.pone.0056535 (PMC3569414; doi:10.1371/journal.pone.0056535)
Supplement: Table S6 — List of the 136 genes from RNA-seq data with structural annotations both in the horse and the human genome. (DOCX) [file pone.0056535.s008.docx]

**Table S6: List of the 136 genes from RNA-seq data with structural annotations both in the horse and the human genome**

| **Gene symbol** | **Gene name** |
| --- | --- |
| *AARSD1* | alanyl-tRNA synthetase domain containing 1 |
| *ACAD9* | acyl-Coenzyme A dehydrogenase family, member 9 |
| *ACADVL* | acyl-Coenzyme A dehydrogenase, very long chain |
| *ACTG1* | actin, gamma 1 |
| *AGPAT6* | 1-acylglycerol-3-phosphate O-acyltransferase 6 (lysophosphatidic acid acyltransferase, zeta) |
| *AHNAK* | AHNAK nucleoprotein |
| *ANKRD11* | ankyrin repeat domain 11; hypothetical protein LOC100128265 |
| *ANKRD17* | ankyrin repeat domain 17 |
| *ANXA2* | annexin A2 pseudogene 3; annexin A2; annexin A2 pseudogene 1 |
| *AP1M1* | adaptor-related protein complex 1, mu 1 subunit |
| *AP2M1* | adaptor-related protein complex 2, mu 1 subunit |
| *API5* | API5-like 1; apoptosis inhibitor 5 |
| *ARNTL* | aryl hydrocarbon receptor nuclear translocator-like |
| *B2M* | beta-2-microglobulin |
| *BBX* | bobby sox homolog (Drosophila) |
| *BRD4* | bromodomain containing 4 |
| *CALCOCO1* | calcium binding and coiled-coil domain 1 |
| *CAPRIN1* | cell cycle associated protein 1 |
| *CAST* | calpastatin |
| *CCNI* | cyclin I |
| *CCR5* | chemokine (C-C motif) receptor 5 |
| *CD40* | CD40 molecule, TNF receptor superfamily member 5 |
| *CDK11A* | similar to cell division cycle 2-like 1 (PITSLRE proteins); cell division cycle 2-like 1 (PITSLRE proteins); cell division cycle 2-like 2 (PITSLRE proteins) |
| *CHD2* | chromodomain helicase DNA binding protein 2 |
| *CLPTM1* | cleft lip and palate associated transmembrane protein 1 |
| *COL14A1* | collagen, type XIV, alpha 1 |
| *COL1A1* | collagen, type I, alpha 1 |
| *COL9A2* | collagen, type IX, alpha 2 |
| *COMP* | cartilage oligomeric matrix protein |
| *COPE* | coatomer protein complex, subunit epsilon |
| *COPS3* | COP9 constitutive photomorphogenic homolog subunit 3 (Arabidopsis) |
| *CRISP2* | cysteine-rich secretory protein 2 |
| *CRISP3* | cysteine-rich secretory protein 3 |
| *CTNNB1* | catenin (cadherin-associated protein), beta 1, 88kDa |
| *CXXC1* | CXXC finger 1 (PHD domain) |
| *CYP17A1* | cytochrome P450, family 17, subfamily A, polypeptide 1 |
| *CYP19A1* | cytochrome P450, family 19, subfamily A, polypeptide 1 |
| *DCTN1* | dynactin 1 (p150, glued homolog, Drosophila) |
| *DNAJC7* | DnaJ (Hsp40) homolog, subfamily C, member 7 |
| *EEF1A1* | eukaryotic translation elongation factor 1 alpha-like 7; eukaryotic translation elongation factor 1 alpha-like 3; similar to eukaryotic translation elongation factor 1 alpha 1; eukaryotic translation elongation factor 1 alpha 1 |
| *EEF1G* | eukaryotic translation elongation factor 1 gamma |
| *EIF3C* | eukaryotic translation initiation factor 3, subunit C |
| *EIF4A1* | similar to eukaryotic translation initiation factor 4A; small nucleolar RNA, H/ACA box 67; eukaryotic translation initiation factor 4A, isoform 1 |
| *EIF4G1* | similar to eukaryotic translation initiation factor 4A; small nucleolar RNA, H/ACA box 67; eukaryotic translation initiation factor 4A, isoform 1 |
| *EIF4G3* | eukaryotic translation initiation factor 4 gamma, 3 |
| *ELL2* | elongation factor, RNA polymerase II, 2 |
| *EWSR1* | similar to Ewing sarcoma breakpoint region 1; Ewing sarcoma breakpoint region 1 |
| *FAU* | Finkel-Biskis-Reilly murine sarcoma virus (FBR-MuSV) ubiquitously expressed |
| *FGFR2* | fibroblast growth factor receptor 2 |
| *FIBP* | fibroblast growth factor (acidic) intracellular binding protein |
| *FILIP1L* | filamin A interacting protein 1-like |
| *GPX1* | glutathione peroxidase 1 |
| *GTF2I* | general transcription factor II, i; general transcription factor II, i, pseudogene |
| *HBP1* | HMG-box transcription factor 1 |
| *HMGCS1* | 3-hydroxy-3-methylglutaryl-Coenzyme A synthase 1 (soluble) |
| *HNRNPUL1* | heterogeneous nuclear ribonucleoprotein U-like 1 |
| *HSP90AA1* | heat shock protein 90kDa alpha (cytosolic), class A member 2; heat shock protein 90kDa alpha (cytosolic), class A member 1 |
| *HSP90AB1* | heat shock protein 90kDa alpha (cytosolic), class B member 1 |
| *HSPA4* | heat shock 70kDa protein 4 |
| *HSPA9* | heat shock 70kDa protein 9 (mortalin) |
| *IL1A* | interleukin 1, alpha |
| *IL4R* | interleukin 4 receptor |
| *IMMT* | inner membrane protein, mitochondrial (mitofilin) |
| *INCENP* | inner centromere protein antigens 135/155kDa |
| *INTS3* | integrator complex subunit 3 |
| *INTS6* | integrator complex subunit 6 |
| *KATNA1* | katanin p60 (ATPase-containing) subunit A 1 |
| *KIF1B* | kinesin family member 1B |
| *LPP* | LIM domain containing preferred translocation partner in lipoma |
| *LTF* | lactotransferrin |
| *MAOA* | monoamine oxidase A |
| *MAP4K1* | mitogen-activated protein kinase kinase kinase kinase 1 |
| *MC1R* | tubulin, beta 3; melanocortin 1 receptor (alpha melanocyte stimulating hormone receptor) |
| *MCM3AP* | minichromosome maintenance complex component 3 associated protein |
| *MDH2* | malate dehydrogenase 2, NAD (mitochondrial) |
| *MGEA5* | meningioma expressed antigen 5 (hyaluronidase) |
| *MLL5* | myeloid/lymphoid or mixed-lineage leukemia 5 (trithorax homolog, Drosophila) |
| *MRPL11* | mitochondrial ribosomal protein L11 |
| *MYH10* | myosin, heavy chain 10, non-muscle |
| *NCAPG2* | non-SMC condensin II complex, subunit G2 |
| *NCKAP1* | NCK-associated protein 1 |
| *NCOR1* | nuclear receptor co-repressor 1 |
| *NHLRC2* | NHL repeat containing 2 |
| *NRBP1* | nuclear receptor binding protein 1 |
| *NRD1* | nardilysin (N-arginine dibasic convertase) |
| *NXF1* | nuclear RNA export factor 1 |
| *OAZ2* | ornithine decarboxylase antizyme 2 |
| *OAZ3* | ornithine decarboxylase antizyme 3 |
| *PA2G4* | proliferation-associated 2G4, 38kDa; proliferation-associated 2G4 pseudogene 4 |
| *PAF1* | Paf1, RNA polymerase II associated factor, homolog (S. cerevisiae) |
| *PC* | pyruvate carboxylase |
| *PDCD11* | programmed cell death 11 |
| *PDCD6IP* | programmed cell death 6 interacting protein |
| *PER2* | period homolog 2 (Drosophila) |
| *PFKP* | phosphofructokinase, platelet |
| *PHIP* | pleckstrin homology domain interacting protein |
| *PIM1* | pim-1 oncogene |
| *PKM2* | similar to Pyruvate kinase, isozymes M1/M2 (Pyruvate kinase muscle isozyme) (Cytosolic thyroid hormone-binding protein) (CTHBP) (THBP1); pyruvate kinase, muscle |
| *PLAUR* | plasminogen activator, urokinase receptor |
| *PLCG2* | phospholipase C, gamma 2 (phosphatidylinositol-specific) |
| *POLDIP2* | polymerase (DNA-directed), delta interacting protein 2 |
| *PRM1* | protamine 1 |
| *PRMT1* | protein arginine methyltransferase 1 |
| *PRNP* | prion protein |
| *PSAP* | prosaposin |
| *PSMC3* | proteasome (prosome, macropain) 26S subunit, ATPase, 3 |
| *PSMD13* | proteasome (prosome, macropain) 26S subunit, non-ATPase, 13 |
| *PSMD2* | proteasome (prosome, macropain) 26S subunit, non-ATPase, 2 |
| *RAB11FIP4* | RAB11 family interacting protein 4 (class II) |
| *RANBP1* | similar to RAN binding protein 1 |
| *RBM26* | RNA binding motif protein 26 |
| *RNF41* | ring finger protein 41 |
| *RPL5* | ribosomal protein L5 pseudogene 34; ribosomal protein L5 pseudogene 1; ribosomal protein L5 |
| *RPS12* | ribosomal protein S12; ribosomal protein S12 pseudogene 4; ribosomal protein S12 pseudogene 11; ribosomal protein S12 pseudogene 9 |
| *RPS4* | ribosomal protein S4 |
| *SAP130* | Sin3A-associated protein, 130kDa |
| *SAPS3* | SAPS domain family, member 3 |
| *SELP* | selectin P (granule membrane protein 140kDa, antigen CD62) |
| *SF3B2* | splicing factor 3b, subunit 2, 145kDa |
| *SGMS1* | sphingomyelin synthase 1 |
| *SMARCA2* | SWI/SNF related, matrix associated, actin dependent regulator of chromatin, subfamily a, member 2 |
| *SOX9* | SRY (sex determining region Y)-box 9 |
| *SPTBN1* | spectrin, beta, non-erythrocytic 1 |
| *STK31* | serine/threonine kinase 31 |
| *STK39* | serine threonine kinase 39 (STE20/SPS1 homolog, yeast) |
| *SUPT6H* | suppressor of Ty 6 homolog (S. cerevisiae) |
| *TAX1BP1* | Tax1 (human T-cell leukemia virus type I) binding protein 1 |
| *TBL3* | transducin (beta)-like 3 |
| *TIMP2* | TIMP metallopeptidase inhibitor 2 |
| *TLE1* | similar to transducin-like enhancer of split 1 (E(sp1) homolog, Drosophila); transducin-like enhancer of split 1 (E(sp1) homolog, Drosophila) |
| *TNP2* | transition protein 2 (during histone to protamine replacement) |
| *UBA1* | ubiquitin-like modifier activating enzyme 1 |
| *UNK* | unkempt homolog (Drosophila) |
| *XPO6* | exportin 6 |
| *ZCCHC6* | zinc finger, CCHC domain containing 6 |
| *ZKSCAN1* | zinc finger with KRAB and SCAN domains 1 |
