# Supplementary material for: Stallion Sperm Transcriptome Comprises Functionally Coherent Coding and Regulatory RNAs as Revealed by Microarray Analysis and RNA-seq
Source: PLoS One. 2013 Feb 11;8(2):e56535. doi: 10.1371/journal.pone.0056535 (PMC3569414; doi:10.1371/journal.pone.0056535)
Supplement: Table S7 — The 82 sperm micro RNAs discovered by RNA-Seq. (DOCX) [file pone.0056535.s009.docx]

**Table S7.** **The 82 sperm micro RNAs discovered by RNA-Seq**; * - miRNAs that fully aligned with equine gene models; bold – miRNA with AC>100

| **Gene Symbol** | **Chromosomal location of mapped tags (EcuCab2); chr:bp** | **Highest AC value** | **Accession No (NCBI)** | **Accession No (miRBase)** |
| --- | --- | --- | --- | --- |
| 1. **MIR1248*** | **19:24793832-24794009** | **536.83** | **NR_032967** | **MI0012839** |
| 1. **MIR34B*** | **7:20101184-20101217** | **336.58** | **NR_032870** | **MI0012741** |
| 1. **MIR34C*** | **7:20101727-20101755** | **336.0** | **NR_032871** | **MI0012742** |
| 1. **MIR223** | **X:48489279-48489317** | **295.64** | **NR_033081** | **MI0012953** |
| 1. **MIR191*** | **16:38001162-38001193** | **160.38** | **NR_032953** | **MI0012825** |
| 1. **MIR1905C** | **7:541073-541099** | **124.94** | **NR_032865** | **MI0012736** |
| 1. MIR761 | 2:7426916-7427239 | 88.9 | NR_032809 | MI0012679 |
| 1. MIR449A* | 21:17151964-17151989 | 81.75 | NR_032979 | MI0012851 |
| 1. MIR764 | X:90696364-90696392 | 73.47 | NR_033113 | MI0012985 |
| 1. MIR1264 | X:90696364-90696392 | 73.47 | NR_033071 | MI0012943 |
| 1. MIR1912 | X:90696364-90696392 | 73.47 | NR_033076 | MI0012948 |
| 1. MIR15B* | 5:15685191-15685222 | 70.84 | NR_032836 | MI0012706 |
| 1. MIR16 | 5:15685191-15685222 | 70.84 | NR_032837 | MI0012707 |
| 1. MIR107A | 14:12370516-12370544 | 60.96 | NR_032933 | MI0012805 |
| 1. MIR25 | 13:8034494-8034522 | 55.24 | NR_032929 | MI0012801 |
| 1. MIR93* | 13:8034494-8034522 | 55.24 | NR_032932 | MI0012804 |
| 1. MIR106B | 13:8034494-8034522 | 55.24 | NR_032925 | MI0012797 |
| 1. MIR490 | 4:90790751-90790781 | 52.1 | NR_032828 | MI0012698 |
| 1. MIR130A | 12:17669064-17669218 | 41.41 | NR_032918 | MI0012790 |
| 1. MIR10B | 18:54601808-54601838 | 41.39 | NR_032965 | MI0012837 |
| 1. MIR199B | 25:31573257-31573327 | 36.38 | NR_033050 | MI0012922 |
| 1. MIR551A | 2:46538535-46538580 | 34.92 | NR_032807 | MI0012677 |
| 1. MIR190B | 5:43518069-43518192 | 32.97 | NR_032840 | MI0012710 |
| 1. MIR15A | 17:21138922-21138948 | 32.96 | NR_032957 | MI0012829 |
| 1. MIR16-2* | 17:21138922-21138948 | 32.96 | NR_032958 | MI0012830 |
| 1. MIR1905A | 1:89368364-89368637 | 29.33 | NR_032783 | MI0012653 |
| 1. MIR150 | 10:19316532-19316601 | 26.95 | NR_032890 | MI0012762 |
| 1. MIR212 | 11:45627317-45627475 | 25.76 | NR_032905 | MI0012777 |
| 1. MIR590 | 13:11685265-11685289 | 25.12 | NR_032931 | MI0012803 |
| 1. MIR1302B-2* | 15:56000399-56000425 | 23.15 | NR_032818 | MI0012688 |
| 1. MIR126 | 25:37331556-37331580 | 23 | NR_033047 | MI0012919 |
| 1. MIR492-1 | 11:21263027-21263053 | 21.03 | NR_032806 | MI0012676 |
| 1. MIR492-2 | 11:21263027-21263053 | 21.03 | NR_032872 | MI0012743 |
| 1. MIR374A | X:55505996-55506019 | 19 | NR_033087 | MI0012959 |
| 1. MIR545 | X:55505996-55506019 | 19 | NR_033109 | MI0012981 |
| 1. MIRLET7D* | 23:54148872-54148896 | 18.39 | NR_032987 | MI0012859 |
| 1. MIRLET7G* | 16:35442185-35442216 | 17.43 | NR_032948 | MI0012820 |
| 1. MIR135B | 5:1885387-1885413 | 17 | NR_032834 | MI0012704 |
| 1. MIR101-2 | 23:26342457-26342490 | 16.82 | NR_032989 | MI0012861 |
| 1. MIR30E | 2:17424933-17424991 | 16.35 | NR_032802 | MI0012672 |
| 1. MIR340 | 14:2544852-2544939 | 16.23 | NR_032939 | MI0012811 |
| 1. MIR1291A | 6:66546402-66546462 | 16.16 | NR_032850 | MI0012721 |
| 1. MIR17 | 17:61793042-61793066 | 16.12 | NR_032959 | MI0012831 |
| 1. MIR18A | 17:61793042-61793066 | 16.12 | NR_032960 | MI0012832 |
| 1. MIR19A | 17:61793042-61793066 | 16.12 | NR_032961 | MI0012833 |
| 1. MIR19B | 17:61793042-61793066 | 16.12 | NR_032962 | MI0012834 |
| 1. MIR20A | 17:61793042-61793066 | 16.12 | NR_032963 | MI0012835 |
| 1. MIR92A | 17:61793042-61793066 | 16.12 | NR_032964 | MI0012836 |
| 1. MIR671 | 4:102818168-102818194 | 15.91 | NR_032831 | MI0012701 |
| 1. MIR632 | 11:40178425-40178456 | 15.55 | NR_032916 | MI0012788 |
| 1. MIR99B | 10:21372086-21372345 | 15.44 | NR_032895 | MI0012767 |
| 1. MIR125A | 10:21372086-21372345 | 15.44 | NR_032888 | MI0012760 |
| 1. MIRLET7E | 10:21372086-21372345 | 15.44 | NR_032887 | MI0012759 |
| 1. MIR1842 | 13:40617024-40617112 | 15.4 | NR_032927 | MI0012799 |
| 1. MIR33B | 11:60068894-60068947 | 15.08 | NR_032910 | MI0012782 |
| 1. MIR29A | 4:85314707-85314730 | 15 | NR_032824 | MI0012694 |
| 1. MIR29B | 4:85314707-85314730 | 15 | NR_032825 | MI0012695 |
| 1. MIRLET7A-2* | 28:42016390-42016416 | 14.91 | NR_033060 | MI0012932 |
| 1. MIR30C* | 2:17432775-17432799 | 14.67 | NR_032801 | MI0012671 |
| 1. MIR140 | 3:19908714-19908741 | 14.62 | NR_032812 | MI0012682 |
| 1. MIR124-2 | 9:20516357-20516380 | 14.46 | NR_032883 | MI0012755 |
| 1. MIR874 | 14:38731755-38731780 | 14.38 | NR_032941 | MI0012813 |
| 1. MIR103 | 22:19036072-19036675 | 14.19 | NR_032982 | MI0012854 |
| 1. MIR10A* | 11:24629538-24629585 | 12.18 | NR_032896 | MI0012768 |
| 1. MIR132* | 11:45639835-45639859 | 12.08 | NR_032898 | MI0012770 |
| 1. MIR26A-2 | 16:47223453-47223877 | 11.22 | NR_032954 | MI0012826 |
| 1. MIR155 | 26:23244624-23244939 | 11.19 | NR_033055 | MI0012927 |
| 1. MIR22 | 11:45380557-45380611 | 10.64 | NR_032906 | MI0012778 |
| 1. MIRLET7F | 23:54160461-54160485 | 10.46 | NR_032988 | MI0012860 |
| 1. MIR220B | 20:3626665-3626691 | 9.85 | NR_032975 | MI0012847 |
| 1. MIR220B-2 | 20:3626665-3626691 | 9.85 | NR_032976 | MI0012848 |
| 1. MIR670 | 12:8508537-8508561 | 9.62 | NR_032923 | MI0012795 |
| 1. MIR125B-2 | 26:15554665-15554690 | 9.46 | NR_033054 | MI0012926 |
| 1. MIR330 | 10:16165787-16165829 | 9.44 | NR_032891 | MI0012763 |
| 1. MIR423* | 11:43782444-43782471 | 9.18 | NR_032912 | MI0012784 |
| 1. MIR192 | 12:25029493-25029595 | 8.94 | NR_032920 | MI0012792 |
| 1. MIR194 | 12:25029493-25029595 | 8.94 | NR_032921 | MI0012793 |
| 1. MIR1282 | 1:145282209-145282600 | 8.78 | NR_032776 | MI0012646 |
| 1. MIR196A | 11:24673751-24673777 | 8.61 | NR_032903 | MI0012775 |
| 1. MIR324 | 11:50248427-50248480 | 7.5 | NR_032908 | MI0012780 |
| 1. MIR128-2 | 18:19481918-19482157 | 6.85 | NR_032966 | MI0012838 |
| 1. MIR711 | 16:38410403-38410428 | 6.73 | NR_032955 | MI0012827 |
