# Supplementary material for: Stallion Sperm Transcriptome Comprises Functionally Coherent Coding and Regulatory RNAs as Revealed by Microarray Analysis and RNA-seq
Source: PLoS One. 2013 Feb 11;8(2):e56535. doi: 10.1371/journal.pone.0056535 (PMC3569414; doi:10.1371/journal.pone.0056535)
Supplement: Table S8 — Correspondence of the RNA-Seq data with the current NCBI gene models for PKM2 , CRISP3 , TNP2 and PRM1 . (DOCX) [file pone.0056535.s010.docx]

**Table S8: Correspondence of the RNA-Seq data with the current NCBI gene models for *PKM2*, *CRISP3*, *TNP2* and *PRM1***

| **Mapped RNA-Seq tag** | **Max AC** | **NCBI Accession** | **Corresponding gene exon** |
| --- | --- | --- | --- |
| **Pyruvate kinase *PKM2* chr1:121089582-121116344** | | | |
| chr1:121099972-121100159 | 297.97 | NM_001159690 | 1 |
|  |  | NM_001143794 | 1 |
| chr1:121105207-121105340 | 240.53 | NM_001159690 | 3 |
|  |  | NM_001143794 | 3 |
| chr1:121097639-121097781 | 185.51 | NM_001159690 | 1- 5’UTR |
|  |  | NM_001143794 | 1- 5’UTR |
| chr1:121105805-121106010 | 180.42 | NM_001159690 | 4 |
|  |  | NM_001143794 | 4 |
| chr1:121106562-121106859 | 176.56 | NM_001159690 | 5 |
|  |  | NM_001143794 | 5 |
| chr1:121108165-121108323 | 102.99 | NM_001159690 | 6 |
|  |  | NM_001143794 | 6 |
| chr1:121092067-121092223 | 94.16 | NM_001159690 | 5’ upstream |
|  |  | NM_001143794 | 5’ upstream |
| chr1:121100717-121100902 | 52.06 | NM_001159690 | 2 |
|  |  | NM_001143794 | 2 |
| chr1:121112623-121112689 | 16.48 | NM_001159690 | 9 |
|  |  | NM_001143794 | 9 |
| **Cysteine-rich secretory protein 3, *CRISP3* chr20:47721075-47745394** | | | |
| chr20:47701763-47701860 | 114.39 | NM_001081874 | none |
| chr20:47702610-47702757 | 36.04 | NM_001081874 | none |
| chr20:47706160-47706240 | 26.1 | NM_001081874 | none |
| **Transition protein 2, TNP2 chr13:33215084-33216483 and Protamine 1, *PRM1* chr13:33206306-33206537** | | | |
| chr13:33206208-33207592 | 1730.25 | NM_001135102 and NM_001083596 | *PRM1* exons 1 and 2 |
| chr13:33215022-33215459 | 68.48 | NM_001135102 and NM_001083596 | *TNP2* cDNA |
| chr13:33209441-33209827 | 39.19 | NM_001135102 and NM_001083596 | *PRM1* 3’ downstream |
| chr13:33208962-33209331 | 34.15 | NM_001135102 and NM_001083596 | *PRM1* 3’ downstream |
| chr13:33216307-33216705 | 30.69 | NM_001135102 and NM_001083596 | *TNP2* cDNA |
| chr13:33211027-33211438 | 21.17 | NM_001135102 and NM_001083596 | *PRM1* 3’ downstream |
| chr13:33203914-33206028 | 15.65 | NM_001135102 and NM_001083596 | *PRM1* 5’ upstream |
| chr13:33203671-33203901 | 14.36 | NM_001135102 and NM_001083596 | *PRM1* 5’ upstream |
| chr13:33198245-33198302 | 12.4 | NM_001135102; NM_001083596 | *PRM1* 5’ upstream |
| chr13:33210585-33210872 | 10.93 | NM_001135102 and NM_001083596 | *PRM1* 3’ downstream |
| chr13:33202982-33203030 | 7.67 | NM_001135102 and NM_001083596 | *PRM1* 5’ upstream |
| chr13:33208269-33208363 | 7.2 | NM_001135102 and NM_001083596 | *PRM1* 3’ downstream |
